# Hfq binds directly to the ribosome‐binding site of IS *10* transposase mRNA to inhibit translation

**DOI:** 10.1111/mmi.12961

**Published:** 2015-03-11

**Authors:** Michael J. Ellis, Ryan S. Trussler, David B. Haniford

**Affiliations:** ^1^ Department of BiochemistryUniversity of Western OntarioLondonOntarioN6A 5C1Canada.

## Abstract

Hfq is a critical component of post‐transcriptional regulatory networks in most bacteria. It usually functions as a chaperone for base‐pairing small RNAs, although non‐canonical regulatory roles are continually emerging. We have previously shown that Hfq represses IS *10*/Tn*10* transposase expression through both antisense RNA‐dependent and independent mechanisms. In the current work, we set out to define the regulatory role of Hfq in the absence of the IS *10* antisense RNA. We show here that an interaction between the distal surface of Hfq and the ribosome‐binding site of transposase mRNA (RNA‐IN) is required for repressing translation initiation. Additionally, this interaction was critical for the *in vivo* association of Hfq and RNA‐IN. Finally, we present evidence that the small RNA ChiX activates transposase expression by titrating Hfq away from RNA‐IN. The current results are considered in the broader context of Hfq biology and implications for Hfq titration by ChiX are discussed.

## Introduction

Hfq is an abundant RNA‐binding protein that acts at the core of complex post‐transcriptional regulatory networks in many bacteria and is critical for stress and virulence responses (Storz *et al*., 2011; Vogel and Luisi, 2011; Sobrero and Valverde, 2012). It is found in at least 50% of sequenced bacteria (Sun *et al*., 2002) and has been predicted to be involved in the regulation of 269 mRNAs in *Escherichia coli* and at least 20% of all genes in *Salmonella* Typhimurium (Guisbert *et al*., 2007; Sittka *et al*., 2008; Ansong *et al*., 2009). Hfq is important for the function of *trans*‐encoded small regulatory RNAs (sRNAs) that base‐pair with partially complementary mRNAs. Hfq binds sRNAs and their partner mRNAs and facilitates intermolecular base‐pairing. This typically affects translation and/or transcript stability. Hfq contains three RNA‐binding surfaces, all of which play a role in promoting base‐pairing between RNAs. The ‘top’ and ‘bottom’ of the toroidal‐shaped Hfq homohexamer are termed the proximal and distal RNA‐binding surfaces respectively. The proximal surface binds short U‐rich sequences typically found in sRNAs while the distal surface binds longer ARN repeats (where A is an adenine, R is a purine and N can be any nucleotide) typically found in mRNAs (Mikulecky *et al*., 2004; Link *et al*., 2009). The proximal surface is proposed to be critical for sRNA stability through interactions with the 3′poly(U) tract following a Rho‐independent terminator (Sauer and Weichenrieder, 2011; Ishikawa *et al*., 2012). The third, less defined surface consists of the outer rim or lateral RNA‐binding surface. This surface connects the proximal and distal RNA‐binding sites. The lateral surface is extremely basic in *E. coli* and may be important for binding internal U‐rich sequences of sRNAs (Sauer *et al*., 2012). One model for Hfq‐catalyzed pairing predicts simultaneous binding of a cognate sRNA and mRNA pair via the proximal and distal surfaces respectively. Hfq‐binding sites are often just outside of RNA pairing sequences so simultaneous binding would tether the RNAs to Hfq while keeping seed regions available for pairing (Panja and Woodson, 2012). The RNAs can then initiate pairing by interacting in either the lateral or proximal surfaces and the RNAs are released as pairing proceeds (Hopkins *et al*., 2011; Hwang *et al*., 2011; Panja *et al*., 2013).

In addition to a role in sRNA‐based regulation, Hfq has been shown to directly affect translation. In the case of *sdhC* mRNA, the sRNA Spot42 recruits Hfq to an AU‐rich region in the translation initiation region (TIR) to inhibit translation. As the Spot42 pairing region in *sdhC* is too far upstream of the TIR to influence translation, it was inferred that stable association of Hfq with *sdhC* was sufficient to compete with 30S ribosomal subunit binding (Desnoyers and Massé, 2012). In another example, Hfq was shown to bind to a translational enhancer in *cirA* mRNA and block translation. Interestingly, in this case, translation repression was relieved by the upstream binding of an sRNA (RyhB) that caused restructuring of the mRNA within the 5′ untranslated region (5′UTR), which ultimately prevented Hfq binding (Salvail *et al*., 2013). Finally, evidence has been presented in two different organisms that Hfq autoregulates expression by binding its own TIR (Vecerek *et al*., 2005; Sobrero and Valverde, 2011). No sRNAs have been implicated in this autoregulatory loop, supporting the contention that Hfq can act directly to inhibit translation. In the above examples, Hfq binding to the TIR of an mRNA is the effector of translational control, in contrast to sRNA‐dependent regulation where the stable sRNA–mRNA duplex is responsible for blocking ribosome binding. Unlike sRNA‐dependent regulation, the role of each RNA‐binding surface of Hfq in direct translational repression is largely unknown. However, Hfq binding to the TIR of target mRNAs would presumably require the distal surface, which preferentially binds purine‐rich sequences such as the Shine–Dalgarno sequence.

**Figure 1:**
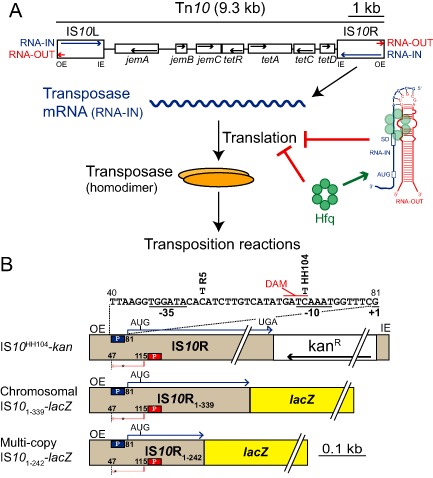
Overview of the Tn*10*/IS *10* system. A. The structure of Tn*10* is shown (Chalmers *et al*., 2000). IS *10* R encodes a functional transposase protein that catalyzes the chemical steps in Tn*10*/IS *10* transposition. In addition to transposase mRNA (RNA‐IN, blue), IS *10* encodes an asRNA (RNA‐OUT, red) that represses transposase translation by blocking ribosome binding. Hfq represses transposase translation by facilitating antisense pairing as well as through an antisense‐independent mechanism. OE and IE are outside and inside ends respectively. B. Schematic of the three IS *10* R constructs used in this work. The promoters for RNA‐IN (pIN) and RNA‐OUT (pOUT) are indicated with blue and red boxes respectively and the transcriptional start sites are shown. RNA‐OUT terminates at nucleotide 47 of IS *10*, which is indicated with a dashed line. The DNA sequence of pIN is shown with the location of two nucleotide changes (R5 and HH104) that each destabilize RNA‐OUT. The DNA adenine methylase (DAM) site, which overlaps with the −10 region, is also shown. The IS *10* ^HH104^‐kan construct consists of a kanamycin resistance gene inserted downstream of the transposase stop codon but upstream of the IE and both translational fusions consist of the indicated portion of IS *10* R fused to codon 10 of *lac* *Z*. In A and B, black arrows indicate the polarity of each open reading frame (ORF).

In addition to its role as an important regulator of endogenous gene expression, Hfq was recently found to suppress Tn*10*/IS*10* transposition in *E. coli* (Ross *et al*., 2010). Tn*10* is a composite transposon containing genes encoding for tetracycline resistance (Fig. 1A). Its component insertion sequence IS*10*‐Right encodes a functional transposase that catalyzes the chemical steps in Tn*10*/IS*10* transposition (Foster *et al*., 1981; Halling *et al*., 1982; Chalmers *et al*., 2000). Expression of IS*10* transposase is regulated by Dam methylation as well as a 69‐nt antisense RNA (asRNA) that is transcribed from the opposite strand of DNA relative to the transposase (Simons and Kleckner, 1983; Roberts *et al*., 1985). The first 35‐nt of this asRNA (RNA‐OUT) is perfectly complementary to the TIR of the transposase mRNA (RNA‐IN), and pairing of these two RNAs inhibits translation by preventing ribosome binding (Fig. 1A) (Ma and Simons, 1990). Antisense control of transposase expression increases with IS*10* copy number, a phenomenon termed ‘multi‐copy inhibition’ (MCI). MCI can be explained by the fact that transposase is a *cis*‐acting protein whereas the asRNA is *trans* acting (Jain and Kleckner, 1993). Accordingly, increasing transposon copy number essentially serves to increase the amount of *trans*‐acting inhibitor while the effective concentration of transposase per element remains constant. Importantly, a single‐copy IS*10* element is not subject to antisense control of transposase expression (Kleckner, 1990).

Hfq was initially linked to Tn*10*/IS*10* transposition when it was found that IS*10* transposition increased in the order of 80‐fold in an *hfq^−^* strain of *E. coli* harboring IS*10* on a multi‐copy plasmid. In contrast, the impact of Hfq deficiency on transposition was greatly reduced (sevenfold increase), but not completely abrogated, when transposition was measured for IS*10* in single copy. These observations were consistent with Hfq contributing to MCI, but also playing a role in down‐regulating IS*10* transposition independent of the MCI pathway (Ross *et al*., 2010). Subsequent work demonstrated that Hfq bound both RNA‐IN and RNA‐OUT *in vitro*, and accelerated the rate of IN‐OUT pairing almost 20‐fold, observations that are consistent with Hfq working through its prototypical RNA pairing pathway to promote the MCI response (Ross *et al*., 2013). It remains to be determined how Hfq regulates Tn*10*/IS*10* transposition when MCI is not in play. However, it has been shown that: (i) in the absence of RNA‐OUT transposase, expression increased sixfold in *hfq^−^*; (ii) there is an Hfq‐binding site in RNA‐IN that overlaps the TIR; and (iii) Hfq status has only a subtle effect on steady‐state RNA‐IN transcript level (Ross *et al*., 2010; 2013). Taken together, it seems likely that Hfq functions in the MCI‐independent pathway by inhibiting translation of RNA‐IN. The goal of the current work was to test this hypothesis and to further characterize how Hfq interacts with RNA‐IN.

## Results

### Antisense‐independent regulation of transposase expression requires the distal surface of Hfq

To gain further insight into how Hfq regulates IS*10* transposase expression (independent of its *cis*‐encoded sRNA), we asked if regulation was maintained when RNA‐binding surfaces of Hfq were mutated. Our expectation was that if a *trans*‐encoded sRNA is involved in this pathway, all three surfaces would be critical for regulation. In particular, we expected the proximal surface to be important for stabilizing any involved sRNAs and the lateral surface for catalyzing pairing with RNA‐IN.

To assess the function of each Hfq‐binding surface in antisense‐independent regulation of transposase expression, we assayed the ability of wild‐type (WT) and mutant forms of Hfq to complement an *hfq^−^* phenotype. We constructed a chromosomal IS*10*_1–339_‐*lacZ* translational fusion with a single bp change (HH104) in the promoter for RNA‐IN, which increases transcription ∼ 100‐fold (Fig. 1B) (Case *et al*., 1988). The HH104 mutation also destabilizes RNA‐OUT; however, a single‐copy transposase‐*lacZ* translational fusion would not normally be regulated by the *cis*‐encoded RNA‐OUT so the net effect of this mutation is to simply increase RNA‐IN expression to detectable levels (Case *et al*., 1989). Expression of transposase‐*lacZ* was measured in an *hfq^−^* strain of *E. coli* harboring plasmids expressing WT Hfq or Hfq deficient in RNA binding at the distal (Y25A), proximal (K56A) or lateral (R17A) surface (Mikulecky *et al*., 2004; Panja *et al*., 2013).

**Figure 2:**
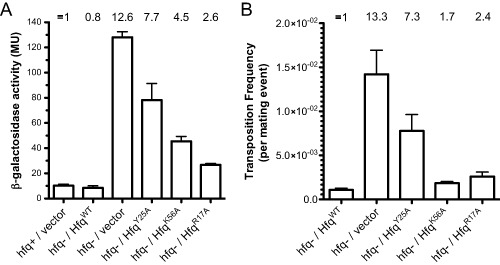
Impact of mutant forms of Hfq on IS *10* transposase expression and transposition. A. Transposase expression was measured in the context of a chromosomal transposase‐*lac* *Z* translational fusion (parent strain DBH298) with the indicated forms of Hfq expressed or in the absence of Hfq expression. The bars show β‐galactosidase activity (Miller units) with standard error of the mean, measured in mid‐exponential phase in LB (*n* = 8). Where indicated, the *hfq^−^* strain (DBH299) was transformed with a low‐copy plasmid encoding Hfq from its native promoter (P3). The mean relative expression observed for each strain is indicated at the top of the graph, where transposase‐*lac* *Z* expression in *hfq^+^* was set at 1. B. Transposition of chromosomal IS *10* ^HH104^‐kan was measured by the conjugal mating out assay (see *Experimental procedures*) in an *hfq^−^* strain (DBH337) transformed with one of the indicated Hfq‐encoding plasmids. The mean relative transposition frequency for each strain is indicated at the top of the graph, where transposition in the presence of Hfq^WT^ was set at 1. Error bars indicate the standard error of the mean for two independent experiments (*n* = 8).

We show in Fig. 2A that transposase‐*lacZ* expression increased almost 13‐fold in the absence of Hfq, and that regulation was fully restored when Hfq^WT^ was expressed from a plasmid. In contrast, none of the Hfq variants were able to fully complement *hfq^−^*, with Hfq^Y25A^ being the most impaired and Hfq^R17A^ functioning the most like WT. Importantly, the reduced function of the mutant proteins cannot be attributed to protein expression as the Hfq levels were not significantly different for WT versus the mutant forms of Hfq (Supporting Information Fig. S1). As the results showed that the integrity of the lateral site is not important for regulation and the integrity of the proximal site is less important than that of the distal site, our data suggested that Hfq repression was sRNA independent.

We also performed the Hfq complementation experiment in the context of an IS*10* transposition assay. As IS*10* transposition frequency is directly proportional to transposase expression (Morisato *et al*., 1983), this experiment allowed us to indirectly measure the effect of Hfq mutations on native transposase expression. Transposition of a single‐copy IS*10* ^HH104^‐kan element (Fig. 1B) in an *hfq^−^* strain of *E. coli* was repressed 13‐fold in the presence of Hfq^WT^ and close to full repression was achieved in the presence of Hfq^K56A^ and Hfq^R17A^ (Fig. 2B). However, in accordance with the expression data (Fig. 2A), Hfq^Y25A^ was the least effective of the mutant Hfq forms in repressing transposition.

It is not clear why the Hfq^K56A^ functioned essentially as WT in the transposition assay (Fig. 2B) while exhibiting a moderate defect in repressing transposase‐*lacZ* expression (Fig. 2A). However, the concordance of the other Hfq variants between the two experiments leads us to conclude that the distal surface is critical for repressing transposase expression in the absence of RNA‐OUT, while the proximal and lateral surfaces are dispensable for regulation.

### The distal surface of Hfq binds the RBS of RNA‐IN *in vitro*

We have previously shown that Hfq binds a 14‐nt A‐rich region of RNA‐IN, which overlaps with the ribosome‐binding site (RBS), as well as an 8‐nt U‐rich region overlapping with codons 5–8 of the transposase coding region (Ross *et al*., 2013). Based on the results from the previous section, we anticipated that the A‐rich‐binding site within the TIR would be the critical site for Hfq regulation as it has the signature of a distal binding site. To further characterize the two Hfq‐binding sites in the 5′ segment of RNA‐IN, we carried out chemical and enzymatic RNA footprinting. Purified WT, Y25A and K56A Hfq were incubated with an RNA corresponding to the first 160‐nt of RNA‐IN (IN‐160) followed by partial digest with lead acetate (Pb^2+^), RNase T1 or RNase V1.

**Figure 3:**
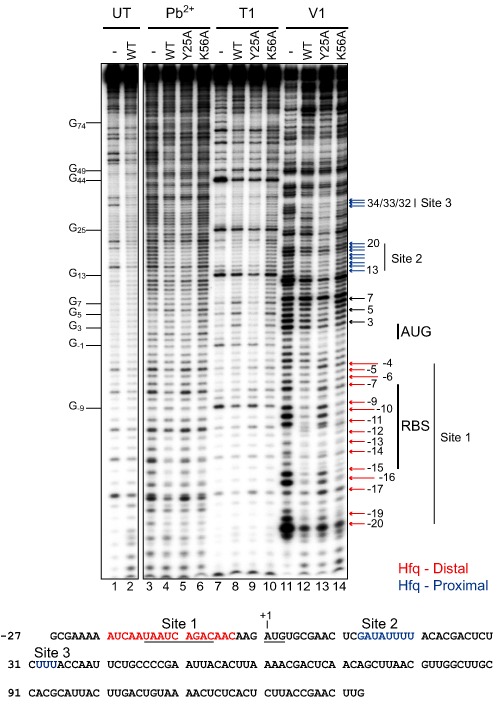
RNA‐IN footprinting with WT and mutant forms of Hfq. 5′^32^P labeled RNA‐IN (40 nM) was incubated with his‐tagged WT, Y25A or K56A Hfq (750 nM Hfq hexamer) before treatment with Pb^2+^, RNase T1 or RNase V1. Untreated RNA was also incubated with or without WT Hfq (lanes 1–2). Nucleotide numbering is relative to the translational start codon (AUG), where the A is position 1. Positions that were protected from cleavage by WT and K56A but not Y25A are indicated with red arrows, while positions protected by WT and Y25A but not K56A are indicated with blue arrows. RNase T1 hypersensitivities are highlighted with black arrows. The nucleotide sequence of the first 160‐nt of RNA‐IN is shown below the gel image with distal and proximal specific‐binding sites indicated in red and blue respectively. The RBS and start codon are underlined.

Within the first 70‐nt of RNA‐IN, three regions of cleavage reagent protection were detected in the presence of Hfq^WT^ (Fig. 3). The most upstream region (relative to the start codon) spans the RBS, extending roughly from nt −20 to −4; hereafter, this site will be referred to as site 1. Additional regions of protection downstream of this include residues 13–20 and 32–34 (hereafter referred to as sites 2 and 3 respectively). Another region spanning nt 3–7 became hypersensitive to RNase T1 and showed reduced RNase V1 cleavage, consistent with Hfq binding inducing a structural transition in this region from dsRNA to ssRNA. In contrast, in reactions with Hfq^Y25A^, site 1 showed greatly reduced protection from both V1 and lead cleavage, consistent with the distal surface of Hfq making contacts with this site. The V1 and lead cleavage pattern in site 1 for Hfq^K56A^ was very similar to that of Hfq^WT^. As Hfq^K56A^ retains a fully functional distal binding surface, this result further supports our conclusion that the distal surface of Hfq contacts site 1.

At sites 2 and 3, the K56A mutation greatly reduced protection from both V1 and lead cleavage whereas the Y25A mutation did not. This is consistent with the proximal surface of Hfq binding both sites 2 and 3.

Finally, the T1 hypersensitivity at positions 3–7 was lost only in the reaction with Hfq^Y25A^. Accordingly, we infer that the structural transition in this region is dependent on the distal face of Hfq binding to site 1.

### Hfq binding to site 1 is critical for regulation of transposase expression *in vivo*

As the distal surface of Hfq is most critical for repression of transposase expression and transposition, our footprinting data suggest that Hfq primarily exerts its regulatory role by binding site 1, which overlaps the RBS of RNA‐IN. We set out to further test this hypothesis by analyzing the impact of nucleotide changes in site 1 and site 2 on Hfq interactions and ultimately transposase expression.

**Figure 4:**
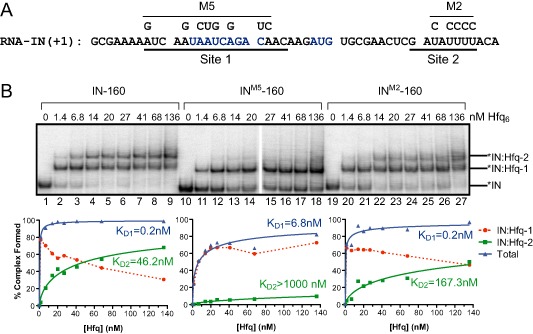
Effect of RNA‐IN mutations on Hfq binding. A. Sequence of the first 50‐nt of RNA‐IN with the RBS and start codon indicated in blue and the sequence of two Hfq‐binding sites (site 1 and site 2) underlined. Nucleotide changes for M5 and M2 mutants are shown. B. Hfq binding to ^32^P‐labeled IN^WT^‐160, IN ^M5^‐160 and IN ^M2^‐160 was measured by EMSA. Binding reactions contained 20 ng μl^−1^ of total yeast RNA, the indicated concentrations of Hfq (reported per hexamer) and ∼ 1 nM RNA‐IN. Band intensities of the representative gel images shown were quantified and the percent complex formed was plotted against Hfq concentration to calculate apparent dissociation constants (K_D_). Hfq‐RNA‐IN interactions for IN:Hfq‐1 and IN:Hfq‐2 are described by K_D1_ and K_D2_ respectively.

Our first objective was to define the relative binding affinity of sites 1 and 2 for Hfq. We show by electrophoretic mobility shift assay (EMSA) in Fig. 4 that in the presence of non‐specific competitor RNA, Hfq^WT^ forms two distinct complexes with RNA‐IN‐160, which we term IN:Hfq‐1 and IN:Hfq‐2. IN:Hfq‐1 was detected at the lowest Hfq concentrations and appears to be converted into IN:Hfq‐2 as Hfq concentrations increased. Apparent K_D_ values were calculated to be 0.2 nM for IN:Hfq‐1 and 46.2 nM for IN:Hfq‐2. Multiple mutations in site 1 were required to reduce formation of IN:Hfq‐1. For example, the M5 mutant contains eight nucleotide changes in site 1 and increased the K_D_ for IN:Hfq‐1 by just over 30‐fold and essentially abrogated the formation of IN:Hfq‐2. In contrast, the site 2 mutant M2, which contains five nucleotide changes, had little impact on IN:Hfq‐1 formation, but increased the K_D_ for IN:Hfq‐2 almost fourfold. Note that because of the manner in which these mutants were identified, we do not know if all the nucleotide changes are necessary for the observed effects (see *Experimental procedures*).

Based on these results, as well as footprinting experiments (Supporting Information Fig. S2), we conclude that IN:Hfq‐1 is formed through Hfq binding to site 1 and IN:Hfq‐2 is formed through Hfq binding to both site 1 and site 2; note we have not looked at the importance of site 3 in IN:Hfq complex formation. Moreover, Hfq binds site 1 with a much higher affinity than it binds site 2, and Hfq binding to site 2 appears to be dependent on Hfq first binding site 1. As formation of IN:Hfq‐2 increased in a concentration‐dependent manner, it is likely that occupancy of site 2 depends on recruitment of a second Hfq hexamer to IN:Hfq‐1, as opposed to the unoccupied proximal surface of an Hfq hexamer bound at site 1 and engaging site 2.

**Figure 5:**
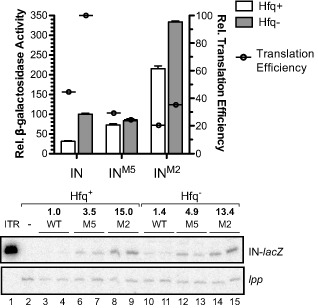
Impact of mutant forms of RNA‐IN on transposase expression in *hfq^+^/hfq^−^* strains. Plasmids encoding WT and mutant forms of a transposase‐*lac* *Z* translational fusion were transformed into *hfq^+^* (DBH107) or *hfq^−^* (DBH12) cells and after growth of transformants to mid‐exponential phase in LB media, β‐galactosidase activity was measured. Error bars show the standard error of the mean for three independent experiments (*n* = 12). RNA was extracted from cells immediately before the Miller assay and RNA‐IN was detected by primer extension (lower panel). *lpp* mRNA was used as a loading control. The relative transcript level from two isolates of each strain was quantified and normalized to WT RNA‐IN in *hfq^+^* (set at 1). The relative translation efficiency for each strain was calculated by dividing Miller units by relative transcript levels (shown as circles on the graph).

We next looked *in vivo* at the impact of disrupting Hfq interactions with sites 1 and 2. To do this, we introduced the M5 and M2 mutations into an IS*10*_1–242_‐*lacZ* translational fusion on a multi‐copy plasmid. A multi‐copy transposase‐*lacZ* translational fusion would normally be highly repressed by the *cis*‐encoded RNA‐OUT. To separate the role of Hfq in direct repression of transposase expression from its role in facilitating antisense pairing, we introduced a single nucleotide mutation (R5) into the promoter region of RNA‐IN, which destabilizes RNA‐OUT while having only a subtle effect on RNA‐IN transcription (Fig. 1B) (Case *et al*., 1988). Based on our earlier results (Fig. 2A and B), our expectation was that the M5 mutation would make IS*10*‐*lacZ* expression insensitive to Hfq status. As the two mutants have multiple nucleotide substitutions, we were concerned that these changes could have indirect effects on transposase expression. Accordingly, we also isolated RNA from cells used in the reporter assays and performed primer extension analysis to monitor steady‐state transcript levels (Fig. 5).

We show in Fig. 5 that both M5 and M2 mutants exhibited reduced Hfq regulation, with the degree of dysregulation being stronger for M5 versus M2. In this reporter setup, there was a 3.2‐fold decrease in transposase expression in the presence versus the absence of Hfq, consistent with Hfq having a negative regulatory role. In contrast, for the M5 reporter, expression levels were essentially the same in *hfq^+^* and *hfq^−^* and for the M2 reporter, expression decreased about 1.5‐fold in the presence of Hfq.

In the above experiment, the ratio of transposase‐*lacZ* expression (β‐galactosidase assay) to the steady‐state level of fusion transcript provides a measure of the translation efficiency. For example, if expression was low and transcript levels were high, this would be indicative of low translation efficiency. The presence of Hfq in cells expressing the WT reporter decreased translation efficiency approximately 2.5‐fold, consistent with Hfq interfering with transposase translation. For the M5 reporter, translation efficiency was greatly reduced compared with the WT reporter, which is not unexpected given that 5 of the 8 nt changes in this construct are in the RBS. Importantly, the translation efficiency did not further decrease in the presence of Hfq. Thus, Hfq is unable to down‐regulate translation when site 1 is mutated. Translation efficiency for the M2 reporter was intermediate to that of the WT and M5 reporters, indicative of site 2 playing a more minor role compared with site 1 in Hfq‐directed repression of translation.

### Hfq blocks 30S ribosome binding to RNA‐IN *in vitro*

**Figure 6:**
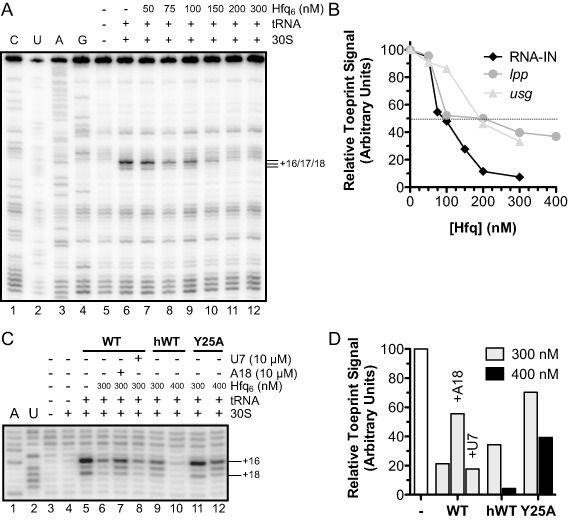
Impact of Hfq on initiation of RNA‐IN translation *in vitro*. A. 30S ribosome binding to RNA‐IN +/− Hfq is shown in a ‘toeprint’ assay (for details, see *Experimental procedures*). Addition of 30S ribosomal subunits and initiator tRNA is indicated by + . The toeprint signal is indicated (+16/+17/+18) with numbering relative to the translational start codon. CUAG refers to sequencing reactions generated from the same RNA used for toeprinting. B. Quantitation of RNA‐IN (A), *lpp* and *usg* (Supporting Information Fig. S3) toeprints. Toeprint signal was normalized to the combined band intensity (+16/+17/+18 for RNA‐IN; +15/+16 for *lpp* and *usg*) in the absence of Hfq, which was set at 100. The dashed line highlights 50% inhibition of the toeprint signal. C. Toeprint analysis of RNA‐IN with distal (A18) and proximal (U7) site‐specific competitor RNAs. In addition, RNA‐IN toeprint signal was also analyzed for reactions containing Y25A versus WT Hfq. Note that for this latter comparison, Hfq contained a C terminal 6x‐His epitope tag. The competitor RNAs alone had no effect on ribosome binding (not shown). D. Band intensities in C were quantified and normalized to the toeprint signal in lane 5 where no competitor or Hfq was added.

Our results thus far show that Hfq inhibits IS*10* transposase expression *in vivo*, and that the most important Hfq‐RNA interaction for this response is between the distal surface of Hfq and site 1, which includes the RBS of RNA‐IN. In addition, results from Fig. 5 are consistent with Hfq down‐regulating IS*10* transposase expression by interfering with IS*10* transposase translation. To further test the hypothesis that Hfq binding to site 1 inhibits RNA‐IN translation, we performed *in vitro* toeprinting assays. We show in Fig. 6A (lane 6) that addition of the 30S ribosomal subunit and initiator tRNA to RNA‐IN resulted in a strong block of reverse transcription at position +16 relative to the RNA‐IN start codon, with minor pauses at nts +17/+18 as has previously been reported (Ma and Simons, 1990). These observations are consistent with a stable translation initiation complex forming on RNA‐IN. When Hfq was added prior to addition of the 30S ribosome and initiator tRNA (lanes 7–12), there was a decrease in the toeprint signal, the magnitude of which was dependent on the Hfq concentration. For example, at an Hfq concentration of 200 nM, where Hfq and RNA‐IN are present at a 1:1 molar ratio, the toeprint signal decreased greater than 90% relative to the signal observed in the absence of Hfq (Fig. 6A and B). In contrast, when the same experiment was performed with *lpp* or *usg* mRNA, inhibition of the toeprint signal was significantly weaker (Supporting Information Fig. S3A). For example, at the same ratio of Hfq:mRNA that gave greater than 90% inhibition for IS*10*, only 50% inhibition was observed for *lpp* and *usg* (Fig. 6B). Although Hfq plays a role in repressing *lpp* expression by stabilizing the sRNA micL (Guo *et al*., 2014), we did not detect *lpp* as an Hfq‐binding mRNA (Fig. 9) and there is conflicting evidence in the literature regarding an interaction between Hfq and *lpp in vivo* (Chao *et al*., 2012; Bilusic *et al*., 2014; Tree *et al*., 2014). However, Hfq does not interact with *usg* mRNA *in vivo* (Beisel *et al*., 2012). Accordingly, the toeprinting results in Fig. 6A are consistent with Hfq acting specifically to block translation initiation in the IS*10* system. We presume that the relatively low level of toeprint inhibition observed in the *lpp* and *usg* experiments is the result of non‐specific interactions between Hfq and components of the 30S ribosome and thus represents ‘background noise’ in the assay.

We reasoned that because the distal surface of Hfq interacts with site 1 of RNA‐IN, then the distal surface of Hfq would be critical for blocking 30S ribosome binding to RNA‐IN. Accordingly, a competitor RNA that is specific for the distal RNA‐binding face of Hfq should relieve the ‘toeprint repression’ afforded by Hfq. We show in Fig. 6C and D that when Hfq was pre‐incubated with A18, a distal site‐binding RNA, prior to its addition to RNA‐IN, the toeprint signal increased approximately 2.5‐fold relative to a control reaction where no competitor was added (compare lanes 6 and 7). Also, if a proximal face‐binding RNA (U7) was used instead of A18, there was no increase in the toeprint signal (compare lanes 6 and 8). We also show in Fig. 6C and D that Hfq^Y25A^ failed to reduce the toeprint signal (compare lanes 9 and 10 with lanes 11 and 12). Taken together, the results in this section show that Hfq can inhibit 30S ribosome binding to RNA‐IN *in vitro* and that this inhibition requires an available distal surface on Hfq. Furthermore, as we have shown that there is a high‐affinity Hfq‐binding site (site 1) that spans the RBS of RNA‐IN and engages the distal binding surface of Hfq, we conclude that Hfq binding to this site is responsible for blocking translation initiation.

### Hfq binds native RNA‐IN *in vivo* and this binding is inhibited by site 1 mutations

**Figure 7:**
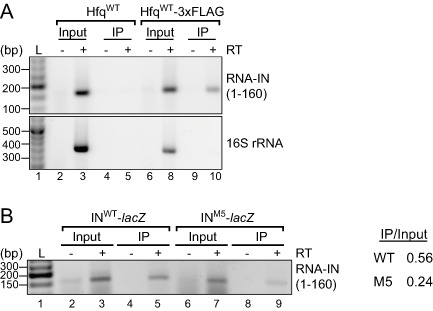
Hfq‐RNA‐IN immunoprecipitation (RIP) assay. A. *hfq^−^* cells containing a chromosomal IS *10* ^HH104^‐kan element (DBH337) were transformed with plasmids expressing Hfq^WT^ (pDH904) or Hfq^WT^‐3xFLAG (pDH909; C‐terminal 3xFLAG tagged Hfq). Hfq was immunoprecipitated (IP) from cell lysates with ANTI‐FLAG ^®^ M2 magnetic beads; untagged Hfq (Hfq^WT^) served as a negative control. The first 160‐nt of RNA‐IN (top panel) or nts 1071–1425 of 16S rRNA were detected by RT‐PCR (see *Experimental procedures*). Samples were analyzed on a 2% agarose gel that was stained with ethidium bromide. No reverse transcription (−RT) controls are shown (lanes 2, 4, 6 and 9). L is a DNA ladder (lane 1). B. *hfq^−^* cells (DBH337) were co‐transformed with Hfq^WT^‐3xFLAG plasmid and a plasmid encoding either WT or M5 transposase‐*lac* *Z*. Hfq RIPs were performed as in A. Band intensities for the input and IP RT‐PCR signal were quantified with an AlphaImager 3400 (Alpha Innotech).

As Hfq is a pleiotropic regulator of gene expression in *E. coli*, there is a concern that any phenotype observed in an *hfq^−^* strain might be the result of dysregulation of a factor under Hfq control. As we are proposing that Hfq binds directly to the 5′UTR of RNA‐IN to block translation, we thought it important to look for Hfq‐RNA‐IN binding *in vivo* where RNA‐IN would have to compete with other cellular RNAs for Hfq binding. We performed an Hfq‐RNA immunoprecipitation experiment (RIP) with *hfq^−^* cells containing a chromosomal copy of IS*10* ^HH104^‐kan and a plasmid encoding FLAG‐tagged Hfq (Fig. 7A). Importantly, this experiment used the same full‐length RNA‐IN as that used in our transposition experiments.

RNA recovered from an Hfq IP was subject to reverse transcription polymerase chain reaction (RT‐PCR) where RNA‐IN was amplified from total cDNA with gene‐specific primers (see *Experimental procedures* and Supporting Information Fig. S4). PCR reactions were then analyzed on an agarose gel. We show that RNA‐IN was strongly enriched in the Hfq IP compared with control reactions (Fig. 7A). Specifically, when cells expressing an untagged Hfq (Hfq^WT^) were subject to RIP, no Hfq was detected in the IP fraction (Supporting Information Fig. S4A) and accordingly no RNA‐IN was detected by RT‐PCR (Fig. 7A). We therefore conclude that RNA‐IN is a bona fide Hfq‐binding partner *in vivo*.

We also performed an Hfq IP with cells containing either the WT or M5 IS*10*‐*lacZ* translational fusion on a multi‐copy plasmid. RT‐PCR analysis of these samples revealed a 2.3‐fold reduction in the recovery of IN^M5^‐*lacZ* compared with IN^WT^‐*lacZ* in the IP. This result is consistent with the Hfq‐binding site within the 5′UTR of RNA‐IN (site 1), providing important determinants for Hfq binding *in vivo* (Fig. 7B).

### ChiX overexpression titrates Hfq away from RNA‐IN

Overexpression of Hfq‐binding RNAs can impinge on other regulatory networks by titrating available Hfq away from other mRNAs and sRNAs (Papenfort *et al*., 2009; Hussein and Lim, 2011; Moon and Gottesman, 2011). Given our finding that RNA‐IN can compete with other cellular RNAs for Hfq binding (Fig. 7), we wondered if induction of an sRNA might increase transposase expression by sequestering Hfq away from RNA‐IN. Although most sRNAs interact with the proximal surface of Hfq, an overexpressed sRNA that can bind the distal surface of Hfq might block Hfq's association with the RBS of RNA‐IN and therefore increase transposase translation by allowing the ribosome to bind. To test this prediction, we screened a library of Hfq‐binding sRNAs to see if any increased transposase‐*lacZ* expression.

We transformed *hfq^+^* cells containing the chromosomal IS*10*_1–339_‐*lacZ* translational fusion with a plasmid expressing 1 of 14 sRNAs (Sgrs, ChiX, RybB, FnrS, MicC, RydC, MgrR, RprA, RyeB, CyaR, MicF, GlmY, MicA and GcvB) or a vector control (Mandin and Gottesman, 2010). Transformants were subject to blue‐white screening on X‐gal plates. Our screen identified a single sRNA, ChiX, which increased IS*10* transposase expression. Notably, the distal surface of Hfq has been previously shown to be important for ChiX stability and Hfq binding *in vivo* (Zhang *et al*., 2013).

**Figure 8:**
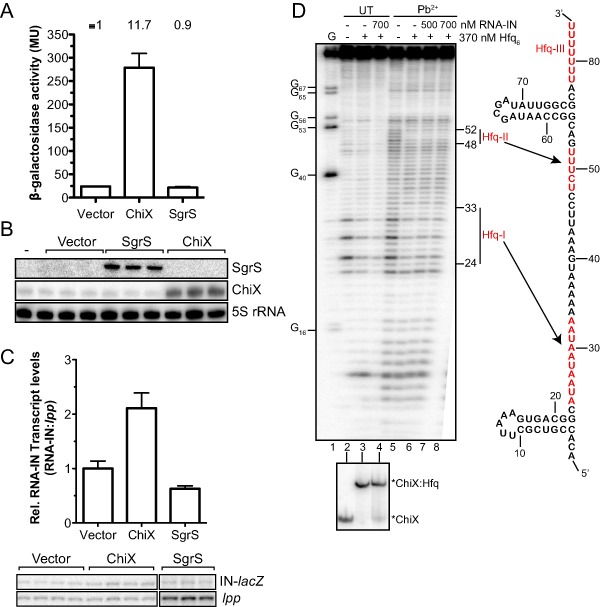
ChiX positively regulates IS *10* transposase translation. A. *hfq^+^* cells containing a chromosomal IS *10*_1–339_‐*lac* *Z* translational fusion were transformed with a plasmid expressing ChiX (pDH765), SgrS (pDH764) or a vector control (pDH763). Transformants were grown to mid‐exponential phase in LB media and β‐galactosidase activity was measured. Error bars show the standard error of the mean for two independent experiments (*n* = 7) and the relative expression is shown above the graph, where transposase‐*lacZ* expression in the presence of vector was set to 1. RNA was extracted immediately before the Miller assay. B. A 3.5 μg of total RNA (three biological isolates) was used for a Northern blot using a 5′^32^P‐labeled oligonucleotide (SgrS) or internally labeled antisense RNA probe (ChiX, 5S rRNA). C. Primer extension analysis of 10 μg of total RNA (four biological isolates) was used to detect RNA‐IN‐*lac* *Z* transcript and *lpp* was analyzed as an internal control. The ratio of IN‐*lac* *Z*:*lpp* was normalized to the vector control and is shown with standard error of the mean as a graph above the gel images. D. 5′^32^P labeled ChiX RNA (100 nM) was incubated with purified Hfq or Hfq and *in vitro* transcribed RNA‐IN before limited cleavage with Pb^2+^. An RNase T1 sequencing lane (G; lane 1) and untreated controls (lanes 2–4) are shown. An aliquot of binding reactions was analyzed on a 6% native polyacrylamide gel (bottom panel). The secondary structure of ChiX is shown (right panel) with three Hfq‐binding sites (Hfq‐I/II/III) highlighted in red.

We next quantified the level of IS*10* transposase up‐regulation by measuring β‐galactosidase activity with overexpression of ChiX. As a control, we included SgrS, as this sRNA did not give a blue colony color in our screen. As shown in Fig. 8A, overexpression of ChiX increased transposase‐*lacZ* expression almost 12‐fold compared with a vector control, while SgrS overexpression had no effect on transposase expression. We also analyzed RNA extracted immediately before the Miller assay by Northern blot and primer extension to measure sRNA induction and transposase‐*lacZ* transcript levels respectively (Fig. 8B and C). Consistent with previous results, a low amount of endogenous ChiX was detected in all samples (Vogel *et al*., 2003; Figueroa‐Bossi *et al*., 2009), but there was a large increase in the presence of the ChiX plasmid (52‐fold). This induction is comparable with that seen as cells transition to stationary phase (Vogel *et al*., 2003). Importantly, ChiX induction resulted in only a twofold increase in transposase‐*lacZ* transcript levels relative to the vector control (Fig. 8C). As ChiX overexpression increased transposase expression 12‐fold while having only a subtle effect on steady‐state transcript levels, we conclude that ChiX increases transposase translation and not transcription or mRNA stability.

**Figure 9:**
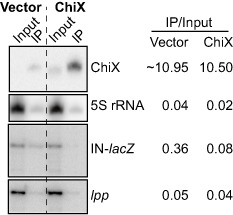
ChiX competes with RNA‐IN for Hfq‐binding *in vivo*. *hfq^−^* cells containing the chromosomal IS *10*_1–339_‐*lac* *Z* translational fusion (DBH299) were co‐transformed with a plasmid encoding Hfq^WT^‐3xFLAG (pDH909) and a plasmid expressing ChiX (pDH765) or vector control (pDH763). Hfq was immunoprecipitated from cell lysates with ANTI‐FLAG ^®^ M2 magnetic beads. Total input RNA (10 μg) or RNA recovered from the IP (0.3 μg) was analyzed by Northern blot for ChiX and the 5S rRNA, or primer extension of RNA‐IN and *lpp*. Band intensities were quantified using ImageQuant.

ChiX may increase transposase translation by one of two mechanisms: (i) ChiX may base‐pair with RNA‐IN to increase ribosome accessibility, as seen in the *rpoS* system (Brown and Elliott, 1997; Soper *et al*., 2010), or (ii) ChiX may bind Hfq with high affinity and block Hfq‐binding to RNA‐IN. We used *in vitro* lead footprinting with 5′labeled ChiX and purified Hfq to define Hfq‐binding sites on ChiX. We also included RNA‐IN in the footprinting reactions to determine if ChiX base‐pairs with RNA‐IN.

In the absence of Hfq, ChiX exhibited high reactivity to lead with the exception of nucleotides 17–22, 56–65 and 70–78, which is consistent with a mostly unstructured RNA containing a 5′stem‐loop and a Rho‐independent terminator (Fig. 8D, lane 5 and right panel). In the presence of Hfq, two regions of reduced lead cleavage consisting of nucleotides 24–33 and 48–52 were observed (Hfq‐I and Hfq‐II, compare lanes 5 and 6). Binding reactions were also analyzed by EMSA (shown beneath footprinting gel image) and showed that Hfq forms a single complex with ChiX sRNA (ChiX:Hfq, lanes 2 and 3).

We also performed footprinting experiments with Hfq‐binding face mutants and ChiX RNA (Supporting Information Fig. S5). We show that the distal surface of Hfq binds Hfq‐I, while binding of Hfq‐II requires an intact proximal surface. In addition, a third Hfq binding site (Hfq‐III) was identified that includes the poly(U) tract following the Rho‐independent terminator, which accordingly interacts with the proximal surface of Hfq.

In the presence of Hfq and a five‐ or sevenfold molar excess of RNA‐IN to ChiX, there were no additional regions protected from lead cleavage (compare lane 5 with 7 and 8). This indicates that ChiX does not base‐pair with the first 160‐nt of RNA‐IN, a conclusion that is also supported by the absence of an additional complex (i.e. ChiX:RNA‐IN binary complex or ChiX:Hfq:RNA‐IN ternary complex) in the EMSA (bottom panel). Addition of RNA‐IN did, however, reduce the lead footprint in the A‐rich Hfq‐binding site of ChiX (compare lane 6 with lanes 7–8) and the amount of ChiX:Hfq complex formed (compare lanes 3 and 4 in the EMSA), consistent with ChiX and RNA‐IN competing for Hfq binding.

Based on our *in vitro* data that RNA‐IN can compete with ChiX for Hfq binding, we performed an RIP in *hfq^−^* cells containing the IS*10*_1–339_‐*lacZ* translational fusion, FLAG‐tagged Hfq and the ChiX overexpression plasmid or a vector control. RNA recovered from the Hfq IP was analyzed directly by Northern blot or primer extension to detect ChiX and IN‐*lacZ* respectively; the 5S rRNA and *lpp* were also analyzed as negative controls (Fig. 9). Overexpression of ChiX resulted in a 4.5‐fold reduction in the amount of RNA‐IN associated with Hfq. This experiment also allowed us to compare the relative binding affinities of ChiX and RNA‐IN for Hfq. In the presence of the vector control, ChiX binds Hfq about 30‐fold better than RNA‐IN *in vivo*. Based on the differences in the amount of RNA analyzed (10 μg input RNA, 0.3 μg IP RNA), we calculated that ChiX was enriched 365‐fold in the Hfq IP, while RNA‐IN was enriched 12‐fold. We presume that the relatively low amount of 5S rRNA and *lpp* mRNA that were detected in the IP represents non‐specific interactions with Hfq *in vivo* or during the IP procedure.

As ChiX was the only sRNA in our screen that titrated Hfq away from RNA‐IN and ChiX is unique among sRNAs in that it contains a distal Hfq‐binding site, we wondered if other RNAs that interact with the distal surface of Hfq would increase transposase expression through an Hfq‐titration mechanism. Most Hfq‐binding mRNAs (including *sodB*, *ptsG* and *maeA*) interact solely with the distal surface of Hfq (Zhang *et al*., 2013). We overexpressed the first 300‐nt of *sodB*, *ptsG* or *maeA* mRNA and measured the impact on transposase expression. Note that the mRNAs were expressed from the same plasmid background as the sRNA overexpression library. Unexpectedly, transposase expression was mostly unaffected by mRNA overexpression (2.4‐fold increase for *sodB*; 1.3‐fold increase for *ptsG* and *maeA*) (Supporting Information Fig. S6).

Together, the above results are consistent with ChiX activating IS*10* transposase translation by titrating Hfq away from RNA‐IN. ChiX does not interact with RNA‐IN but does bind Hfq with high affinity and specificity and can compete with RNA‐IN for Hfq binding *in vitro* and *in vivo*. Because overexpression of mRNAs containing a distal‐binding site did not affect transposase expression, we think it likely that the ability of ChiX to up‐regulate transposase expression is due to the fact that this sRNA possesses both distal and proximal Hfq‐binding sites.

## Discussion

### Direct repression of IS *10* translation by Hfq

Hfq typically regulates translation by catalyzing pairing of an sRNA to the TIR of an mRNA. In the simplest model, Hfq simultaneously binds an sRNA and cognate mRNA near or overlapping the pairing sequences (also known as seed regions), and as pairing proceeds, Hfq is released from the sRNA–mRNA duplex, whereupon it can catalyze additional pairing reactions (Fender *et al*., 2010; Hwang *et al*., 2011; Panja and Woodson, 2012; Tree *et al*., 2014). The role of each Hfq‐binding surface in sRNA‐dependent regulation has been studied extensively. A study of seven different sRNA–mRNA pairs found that the proximal surface (in particular lysine 56) is critical for Hfq chaperone activity and sRNA stability (Zhang *et al*., 2013). The lateral surface of Hfq also interacts with sRNAs and is important for sRNA stability as well as providing a favorable surface for nucleating pairing (Sauer and Weichenrieder, 2011; Sauer *et al*., 2012; Panja *et al*., 2013); however, this surface of Hfq is dispensable for some systems. Unlike the proximal surface, the distal RNA‐binding site is not an absolute requirement for sRNA‐dependent regulation and in some cases may simply serve as a way to tether Hfq to target mRNAs (Sauer, 2013; Zhang *et al*., 2013). The role of each RNA‐binding surface of Hfq in sRNA‐independent regulation has not been studied.

We first measured the impact of Hfq‐binding face mutations on IS*10* transposase expression and transposition. The finding that the proximal surface is only partially required for regulation suggested that Hfq is functioning independent of a *trans*‐encoded sRNA. The moderate effect of the K56A mutation on Hfq is likely a result of reduced binding to site 2 in RNA‐IN, which is supported by decreased regulation in the presence of the M2 mutations. Additionally, the lateral surface was not required for repressing transposase expression or transposition. Alone, the R17A phenotype does not exclude sRNA‐dependent regulation. In the case of *rpoS*, multiple mutations to the lateral surface resulted in the strongest decrease in sRNA‐mediated activation of *rpoS* expression (Panja *et al*., 2013). Additionally, the lateral surface was only important for about half of the sRNA/mRNA pairs tested previously (Zhang *et al*., 2013). However, a dispensable lateral surface is consistent with sRNA‐independent regulation in the IS*10* system. Unlike most sRNA‐dependent regulation, the distal surface is critical for repressing transposase translation and mutations that block the interaction between the TIR of RNA‐IN and the distal site on Hfq strongly de‐repressed transposase expression *in vivo*. Additionally, an available distal surface on Hfq was required for blocking 30S ribosome binding to RNA‐IN *in vitro*. We therefore suggest that sRNA‐independent regulation by Hfq requires mRNA binding through the distal surface and not the proximal or lateral surfaces.

For Hfq to be an effective direct inhibitor of translation, we think some very specific requirements must be met. Firstly, there needs to be an Hfq‐binding site in the TIR of an mRNA. Interestingly, a recent survey of RNA sequences bound by Hfq *in vivo* included repeated trinucleotide motifs (ARN) that were frequently associated with the Shine–Dalgarno sequence. Notably, 18% of all Hfq‐associated mRNAs contained Hfq‐binding sites located within the TIR (Tree *et al*., 2014). Secondly, the Hfq‐binding site within the TIR should be a high affinity site to ensure *in vivo* binding. Thirdly, there must be sufficient available Hfq to act stoichiometrically on TIRs. Given that Hfq is a highly expressed protein, this third factor might not appear to be a limitation. However, there is growing evidence that despite being highly abundant (5000–10 000 hexamers per cell) (Kajitani *et al*., 1994; Ali Azam *et al*., 1999; Argaman *et al*., 2012), the amount of unbound Hfq at any given time might in fact be limiting for RNA binding (Hussein and Lim, 2011; Moon and Gottesman, 2011). Hfq has a large number of specific mRNA and sRNA targets and may also be sequestered through mostly non‐specific DNA interactions (Azam and Ishihama, 1999; Updegrove *et al*., 2010). Accordingly, for translational repression where a sustained interaction with an mRNA is required to block 30S ribosomal subunit binding, it is likely critical that Hfq binds with extremely high affinity to the TIR, which might compensate for limited availability of Hfq. We found this to be the case in the IS*10* system as Hfq bound the TIR with an affinity of approximately 0.2 nM. This represents one of the highest affinity interactions between Hfq and an mRNA [cf. ompA 1 nM, ompC 0.9 nM, ompF 4 nM (Fender *et al*., 2010), sodB 0.3 nM (Geissmann and Touati, 2004) and rpoS ∼50 nM (Soper *et al*., 2011; Peng *et al*., 2014)]. Moreover, we found that a moderate increase in expression of ChiX sRNA was sufficient to de‐repress transposase expression. Given our evidence that (i) ChiX possesses a distal Hfq‐binding site; (ii) its overexpression did not substantially influence RNA‐IN steady‐state levels; (iii) ChiX does not base‐pair with RNA‐IN; and (iv) ChiX overexpression reduces the amount of RNA‐IN bound by Hfq *in vivo*, we think the results of the ChiX overexpression experiment are most easily explained by a ChiX‐Hfq titrating mechanism. That is, despite the high affinity of site 1 in RNA‐IN for Hfq, moderate overexpression of ChiX was sufficient to deplete the pool of available Hfq such that there were insufficient amounts to repress RNA‐IN translation. The implications of Hfq titration by ChiX are discussed below.

### Hfq regulation of IS *10* in single and multi‐copy

**Figure 10:**
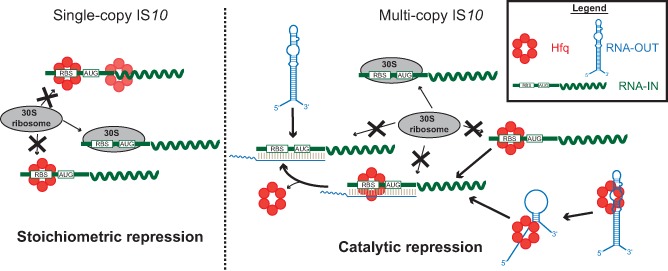
Model for a dual role of Hfq in repressing IS *10* transposase translation. When IS *10* is present in single copy (left side), transposase expression is not subject to antisense control by RNA‐OUT. Hfq binding to the RBS in RNA‐IN represses translation by preventing ribosome binding (‘stoichiometric’ repression). Multi‐copy IS *10* is subject to antisense control by increased concentrations of RNA‐OUT (right panel). Hfq may still participate in stoichiometric repression but also facilitates antisense pairing in a ‘catalytic’ manner. Hfq binding to RNA‐IN and RNA‐OUT alters RNA secondary structure (not shown for RNA‐IN), exposing sequences involved in pairing.

All of the *in vivo* experiments reported in this work were performed under conditions where the naturally occurring antisense RNA (RNA‐OUT) was not produced. This was meant to mimic a situation where IS*10* is in single copy and the antisense RNA has little to no effect on transposase expression (Kleckner, 1990). The HH104 mutation was introduced to the single‐copy IS*10* elements to increase transposase transcription to detectable levels, and although this mutation also eliminates the small amount of cis‐encoded RNA‐OUT, there would be little antisense control to begin with. We propose that under these conditions, Hfq acts in a stoichiometric manner to limit RNA‐IN translation (Fig. 10). However, when IS*10* is present on a multi‐copy plasmid, stoichiometric action of Hfq on RNA‐IN might not be sufficient to limit translation because of the increase in the number of RNA‐IN transcripts. In support of this, when we compared Hfq regulation of a chromosomal IS*10*‐*lacZ* fusion with a high‐copy translational fusion, the extent of Hfq‐mediated repression was attenuated from 13‐ to 3‐fold (Figs 2A and 5). With a high‐copy IS*10* element, the MCI pathway comes into play and RNA‐OUT becomes an important negative regulator of RNA‐IN translation and stability (Case *et al*., 1990). We have previously shown that Hfq binds RNA‐OUT and promotes its restructuring to expose sequences important for RNA‐IN pairing (Ross *et al*., 2013). As Hfq facilitates RNA‐IN:OUT pairing in a catalytic cycle, we think that when IS*10* is in multi‐copy, this mechanism would predominate over the ‘stoichiometric’ inhibition pathway. We eliminated RNA‐OUT from the multi‐copy IS*10*_1–242_‐*lacZ* translational fusion so we could study antisense‐independent regulation by Hfq, and our results show that stoichiometric repression can still occur albeit regulation is weaker for multi‐copy IS*10*. We therefore think that Hfq is a negative regulator of IS*10* regardless of copy number. This model may also explain previous results where it was found that Hfq is a stronger negative regulator of transposition when IS*10* is present in multi‐copy compared with single copy (Ross *et al*., 2010). Our model would predict that in this scenario, Hfq is repressing predominantly by a catalytic mechanism involving Hfq‐mediated RNA‐IN:OUT pairing, which presumably is more efficient than the stoichiometric repression that is confined to the single‐copy situation.

This dual model for Hfq repression may be applicable to other members of the Hfq regulon. In the absence of a cognate sRNA, we suggest that Hfq would repress translation of mRNAs containing an Hfq‐binding site in the TIR by a stoichiometric mechanism. The strength of this regulation would be governed primarily by the affinity of Hfq for that site relative to other cellular mRNAs. This model might also explain the conflicting data concerning regulation of *ompA* expression. Work in the Bläsi lab suggested that Hfq directly represses *ompA* translation by binding the TIR in a manner analogous to that described here for IS*10* (Vytvytska *et al*., 2000). This is supported by several surveys of Hfq‐binding mRNAs that have identified *ompA* as an Hfq‐binding mRNA (Zhang *et al*., 2003; Sittka *et al*., 2008; Tree *et al*., 2014). The characterization of the stationary‐phase sRNA MicA by the Wagner lab suggested that the primary role of Hfq in regulating *ompA* expression was to promote sRNA–mRNA pairing. Our model would combine both mechanisms and suggest that Hfq exerts some basal repression of *ompA* translation that is strengthened in stationary phase by promoting MicA pairing with *ompA*. Additionally, our model is applicable to the *sdhC* system where Hfq had an sRNA‐independent effect on expression that is presumably a result of direct repression of translation (Desnoyers and Massé, 2012).

### Hfq titration by the sRNA ChiX

Induction of Hfq‐binding sRNAs can impinge on other Hfq‐dependent post‐transcriptional networks. In the simplest model, induction of an sRNA would provide enough sRNA molecules to bind all available Hfq and even compete with other Hfq‐binding RNAs. Hfq titration was first proposed as a mechanism for OxyS repression of *rpoS* expression, which was later verified (Zhang *et al*., 1998; Moon and Gottesman, 2011). ChiX overexpression also resulted in increased *rpoS*‐*lacZ* expression, and this effect was proposed to be a result of Hfq titration (Mandin and Gottesman, 2010). Additionally, overexpression of ArcZ in *Salmonella* was shown to have a pleiotropic effect on gene expression (altering expression of 757 genes) in part by decreasing the number of mRNAs bound to Hfq as well as specific competition with the sRNAs CyaR and InvR (Papenfort *et al*., 2009). Our studies with ChiX overexpression have provided another example of an sRNA sequestering sufficient amounts of Hfq to produce a biological effect: de‐repression of RNA‐IN translation.

ChiX (previously named SroB, RybC and MicM) is a negative regulator of genes involved in chitobiose utilization. ChiX is constitutively expressed but its levels increase substantially in stationary phase (Vogel *et al*., 2003; Figueroa‐Bossi *et al*., 2009; Papenfort and Vogel, 2014). The fact that ChiX was the only sRNA of 14 screened to have an impact on expression of RNA‐IN under antisense‐independent conditions fits fully with our model of stoichiometric inhibition resulting from Hfq binding the TIR of RNA‐IN (site 1) through its distal binding site. ChiX is somewhat unique among *E. coli* sRNAs, as (according to our footprinting data) it possesses both distal and proximal Hfq‐binding sites. Moreover, in a recent study that compared the ability of a set of sRNAs to compete for Hfq binding, it was established that ChiX was at the top of the hierarchy, while SgrS was at the bottom. This same work also showed that the A‐rich region of ChiX that we designated Hfq‐I interacts with the distal surface of Hfq and provides important determinants for Hfq competition (Małecka *et al*., 2015). This fits fully with our data showing that ChiX overexpression titrated Hfq away from RNA‐IN while SgrS did not.

ChiX is unique among sRNAs as it acts catalytically to repress its target mRNA (*chiP*, previously known as *ybfM*), and ChiX levels are regulated by an ‘anti‐sRNA’, *chbBC* (Overgaard *et al*., 2009). It is therefore unexpected that ChiX levels would increase so dramatically during stationary phase. Given the current work, it is tempting to speculate that ChiX has a yet unidentified role during the transition to stationary phase; notably, ChiX constitutes 24–26% of Hfq‐bound sRNAs during early stationary phase (Chao *et al*., 2012) . Based on ChiX's distinct interaction properties with Hfq, it is worth considering the possibility that ChiX expression could influence the entire Hfq regulon during the transition to stationary phase.

## Experimental procedures

### Bacterial strains, phage, plasmids and oligonucleotides

All bacterial strains, phage and plasmids used in this study are listed in Supporting Information Table S1 and oligonucleotides are listed in Supporting Information Table S2.

For mating out experiments, DBH33 was lysogenized with λDBH504 to create DBH331 (*hfq^+^*). P1 transduction was then performed to convert DBH331 to DBH337 (*hfq^−^*). λDBH504 was created by crossing IS*10* ^HH104^‐kan from pNK1223 onto λNK1039 in DBH60; kan^R^ lysogens were selected by replica plating and then phage stocks (λDBH504) were prepared from these lysogens. For β‐galactosidase assays with chromosomal IS*10* ^HH104^‐*lacZ*, DBH107 was lysogenized with λRS271 (obtained from DBH90 via spontaneous phage release) to create DBH287. DBH287 was subjected to recombineering (details available upon request) to remove the G8 mutation creating DBH298. P1 transduction was then performed to convert DBH298 (*hfq^+^*) to DBH299 (*hfq^−^*).

The Hfq expression plasmids used for complementation experiments were made by amplifying the *hfq* gene (including the P3 promoter) from pDH700, pDH701 and pDH713 (Ross *et al*., 2013) with primers oDH518 and oDH519. The PCR product was digested with HaeIII and cloned into the XmnI/ScaI sites of pACYC184. The R17A mutation was first introduced into pDH700 by overlap PCR using primers oDH518, oDH519, oDH520 and oDH521. The PCR product was cloned into the XbaI/HindIII sites of pDH700 to make pDH874, and Hfq^R17A^ was then subcloned into pACYC184 as above. Hfq was amplified from pDH904 with oDH184 and oDH479 to add a C‐terminal 3xFLAG tag and this amplicon was cloned directly into XmnI/ScaI‐digested pACYC184.

The multi‐copy IS*10*‐*lacZ* translational fusion is a derivative of pNK2974 (Jain, 1995). First, IS*10* was amplified with primers oDH502 and oDH503 and this amplicon was cloned into the EcoRI/HindIII sites of pNK2974 to produce pDH858, which contains the first 242‐nt of IS*10*R fused in frame to codon 10 of *lacZ*. All subsequent mutations were introduced into pDH858 using overlap PCR with primers oDH505 and oDH13 and the relevant mutagenic primers: R5 (oDH506, oDH507), M2 (oDH498, oDH499) and M5 (oDH508, oDH509). PCR products were digested with EcoRI and HindIII and cloned into the same sites in pDH858. M5 and M2 were originally identified as increased expression mutants in a transposase expression screen. The transposase gene used was derived from a library of sequences generated by mutagenic PCR.

Plasmids overexpressing *sodB*, *ptsG* and *maeA* were constructed as previously described (Zhang *et al*., 2013). MC4100 genomic DNA served as a template for PCR with the following primers: oDH558 and oDH559, *sodB*; oDH560 and oDH561, *ptsG*; and oDH562 and oDH563, *maeA*. PCR products were digested with AatII and EcoRI and cloned into the same sites of pDH765.

### Hfq footprinting and EMSA

*In vitro* transcription templates were generated by PCR using plasmids pDH866 (IN‐160), pDH868 (IN^M2^‐160) and pDH875 (IN^M5^‐160) and primers oDH515 (IN‐160 and IN^M2^‐160) or oDH510 (IN^M5^‐160) with oDH199. RNA‐IN was generated by *in vitro* transcription and internally labeled with [α^32^P]‐UTP (for EMSA) or 5′labeled with [γ^32^P]‐ATP (for footprinting) as previously described (Ross *et al*., 2013). WT Hfq was purified by heat treatment and poly(A) affinity purification, and his‐tagged Hfq variants were purified by Ni^2+^‐IMAC as previously described (Ross *et al*., 2013). RNA footprinting was performed as previously described (Ross *et al*., 2013) except that reactions were in 1X RNA Structure Buffer (Ambion, Life Technologies) and Pb^2+^ footprinting used 10 mM Lead(II)Acetate (Sigma‐Aldrich) for 3 min at ambient temperature, which was stopped by addition of ethylenediaminetetraacetic acid (EDTA) to a final concentration of 50 mM. Following ethanol precipitation, RNA footprinting samples were resuspended in denaturing load dye [95% (v/v) formamide, 0.5× Tris‐borate EDTA (TBE), 3% (w/v) xylene cyanol] and resolved on a 10% polyacrylamide gel containing 7 M urea. EMSA was performed essentially as described (Ross *et al*., 2013) except that binding reactions included 20 ng μl^−1^ total yeast RNA (Ambion, Life Technologies). Apparent dissociation constants were determined as previously described (Ross *et al*., 2013).

The ChiX *in vitro* transcription template was generated with primers oDH528 and oDH529 with a genomic DNA template. Lead footprinting was performed as above. A 3.5 μl aliquot of ChiX, ChiX‐Hfq or ChiX‐Hfq‐RNA‐IN (700 nM) was removed from binding reactions, mixed with native load dye [20 mM Tris‐HCl, pH 7.5, 10 mM dithiothreitol (DTT), 100 mM KCl, 30% glycerol (v/v), 0.05% bromophenol blue (w/v)] and resolved on a 6% polyacrylamide TBE gel.

### β‐galactosidase assays

Cells were grown in Luria broth (LB, Miller; Difco) supplemented (where necessary for plasmid selection) with ampicillin (100 μg ml^−1^) and tetracycline (10 μg ml^−1^). Saturated overnight cultures were used to seed subcultures (1:40 dilution), which were grown to mid‐log phase (OD_600_ = 0.4–0.6). IPTG (1 mM) was added to the subculture to induce sRNA expression. The Miller assay was performed as previously described (Ross *et al*., 2010).

### Conjugal mating out assay

The mating out assay was performed essentially as previously described (Ross *et al*., 2010). Plasmids encoding the Hfq variants (pDH904, pDH905, pDH906 and pDH907) or a vector control (pDH900) was transformed into the donor strain DBH337 (*hfq^−^*; contains IS*10* ^HH104^‐kan lysogen) and plated on M9‐glucose supplemented with thiamine (1 μg ml^−1^), arginine (40 μg ml^−1^), kanamycin (50 μg ml^−1^) and tetracycline (15 μg ml^−1^). Donor colonies were grown overnight to saturation in LB supplemented with kanamycin (50 μg ml^−1^) and tetracycline (15 μg ml^−1^) and the recipient strain (HB101) was grown in LB supplemented with streptomycin (150 μg ml^−1^). Donor and recipient strains were subcultured in LB without antibiotics, and then grown and mixed for mating as previously described (Ross *et al*., 2010). Mating was stopped by vigorous vortexing after 1 h and 1 ml of mating mixture was washed and then serially diluted in saline [0.85% (w/v) NaCl]. Cells were plated on M9‐glucose supplemented with thiamine, leucine (40 μg ml^−1^) and streptomycin (150 μg ml^−1^) or streptomycin plus kanamycin (50 μg ml^−1^) for ‘total exconjugates’ and ‘hops’ respectively. The transposition frequency was calculated by dividing the number of Sm^R^Kan^R^ colonies by Sm^R^ colonies (‘hops’ per ‘exconjugate’).

### RNA extraction and primer extension analysis

Cells were grown in LB supplemented with ampicillin (100 μg ml^−1^) to OD_600_ = 0.6 at which time 600 μl of cells was added to 300 μl of RNA lysis buffer [1.5% (w/v) sodium dodecyl sulfate (SDS), 300 mM sodium acetate, 30 mM EDTA] and boiled for 1 min. Samples were chilled on ice for 30 s and then sequentially extracted twice with acid phenol (pH 4.3), once with phenol:chloroform:isoamyl alcohol (25:24:1) and once with 2‐butanol followed by ethanol precipitation. Residual genomic DNA was removed with TURBO DNase (Ambion, Life Technologies) prior to primer extension analysis. Five or 10 μg of total RNA (Figs 5B, 8C and 9 respectively) was subject to primer extension analysis with 5′^32^P‐labeled oDH511 (RNA‐IN) and oDH482 (*lpp*) and SuperScript III (Invitrogen) according to the manufacturer's instructions.

### Toeprinting

The 30S ribosomal subunit was prepared as previously described (Fechter *et al*., 2009). *In vitro* transcribed RNA (2 pmol) was annealed to 5′^32^P‐labeled oDH511 (RNA‐IN), oDH482 (*lpp*) and oDH555 (*usg*) in toeprint buffer (10 mM Tris‐acetate, pH 7.6, 1 mM DTT, 100 mM potassium acetate) by heating to 95^o^C for 1 min followed by snap‐cooling on ice for 2 min. While on ice, magnesium acetate was added to a final concentration of 10 mM and dNTPs to 0.5 mM. Reactions were then incubated at 37^o^C for 5 min. Hfq (0.5–4 pmol of hexamers) or buffer was added to reactions, which were incubated for another 15 min at 37^o^C, followed by addition of 30S ribosome (3.6 pmol for RNA‐IN and *usg*; 2.7 pmol for *lpp*) and incubation at 37^o^C for 5 min. Initiator fMet‐tRNA (10 pmol; Sigma‐Aldrich) was added and reactions were incubated for a further 15 min before addition of 200 U of SuperScript II (Invitrogen) and a final incubation of 10 min at 37^o^C. Reactions were stopped by addition of 100 μl of stop solution [50 mM Tris‐HCl, pH 7.5, 0.1% SDS (w/v), 10 mM EDTA] followed by phenol:chloroform:isoamyl alcohol extraction and ethanol precipitation. Samples were resuspended in denaturing load dye [95% (v/v) formamide, 0.5× TBE, 3% (w/v) xylene cyanol] and resolved on a 10% polyacrylamide gel containing 7 M urea. Dried gels were exposed to a phosphorimager storage screen, imaged with a Storm imager and quantitated with ImageQuant (GE Healthcare).

### Hfq‐RNA immunoprecipitation (RIP)

DBH337 (*hfq^−^*; contains IS*10* ^HH104^‐kan lysogen) was transformed with plasmids expressing untagged Hfq (pDH904) or Hfq with a C‐terminal 3xFLAG tag (pDH909). Cells were grown to mid‐exponential phase (OD_600_ = 0.5) in LB supplemented with tetracycline (15 μg ml^−1^) at which point 50 OD_600_ of cells was collected by centrifugation and washed once in Tris‐buffered saline (TBS; 50 mM Tris‐HCl, pH 7.4, 150 mM NaCl) and resuspended in 400 μl of lysis buffer (20 mM Tris‐HCl, pH 8.0, 150 mM KCl, 1 mM MgCl2) with 40 U of RNasin (Promega) and 2 U of TURBO DNase (Ambion, Life Technologies). Cells were mixed with 400 μl of zirconia/silica beads (0.1 mm, BioSpec) and lysed by vortexing (30 s burst, 30 s ice; 10 cycles), after which 800 μl of lysis buffer was added followed by centrifugation (10 min, 13 500 x g, 4^o^C). An aliquot of the cleared lysate (100 μl) was phenol extracted and ethanol precipitated (‘input RNA’). ANTI‐FLAG^®^ M2 magnetic beads (25 μl packed resin; Sigma‐Aldrich) were added to 800 μl of cleared lysate and samples were incubated at 4^o^C with rotation for 4 h. Beads were washed five times with 1 ml of lysis buffer, and resuspended in 400 μl of lysis buffer. Hfq‐bound RNA (‘IP RNA’) was recovered by phenol extraction and ethanol precipitation. Following precipitation, residual DNA was removed with TURBO DNase (Ambion, Life Technologies) and samples were ethanol precipitated and finally resuspended in diethylpyrocarbonate (DEPC)‐treated ddH_2_O. RNA concentration was determined with a NanoPhotometer (Implen).

For the RIP with WT and mutant IS*10*‐*lacZ* (Fig. 7B), DBH12 was transformed with pDH909 (Hfq‐3xFLAG) and pDH866 (IN^WT^‐*lacZ*) or pDH875 (IN^M5^‐*lacZ*). Cells were grown in LB supplemented with tetracycline and ampicillin (100 μg ml^−1^) and IP was performed as above.

The RIP with ChiX overexpression used DBH337 (*hfq^−^*; contains IS*10* ^HH104^‐kan lysogen) transformed with pDH909 (Hfq‐3xFLAG) and pDH765 (ChiX) or pDH763 (vector). IP was performed as above. Total input RNA (10 μg) or RNA recovered from the IP (0.3 μg) was analyzed directly by Northern blot (ChiX and 5S rRNA) or primer extension (RNA‐IN and *lpp*).

### RT‐PCR

An RNA adapter (oDH486; 100 pmol) was ligated to input (10 μg) or IP (1 μg) RNA using T4 RNA ligase. Adapter‐ligated RNA was purified with an RNeasy Mini Kit (Qiagen), eluted in 30 μl of DEPC ddH_2_O, and 10 μl of RNA was converted to cDNA using an adapter‐specific primer (oDH352) and SuperScript III (Invitrogen) according to the manufacturer's instructions. Enzyme was omitted for the no reverse transcription controls (−RT). Reverse transcription reactions were purified with a PCR purification kit (Qiagen) and eluted in 50 μl of ddH_2_O. The first 160‐nt of RNA‐IN was detected by 28 cycles of PCR using 4 μl of cDNA as a template and primers oDH199 and oDH483 (WT) or oDH517 (M5). A portion of the 16S rRNA (nt 1071–1425) was detected by 18 cycles of PCR using primers oDH204 and oDH205. PCR reactions were analyzed on a 2% agarose TBE gel.

### Northern blot

A 3.5 μg of total RNA (Fig. 8B) was denatured in load dye [95% (v/v) formamide, 0.5× TBE, 3% (w/v) xylene cyanol] and then separated on a 10% polyacrylamide gel containing 7 M urea. RNA was electroblotted to a Hybond‐N nylon membrane (GE Healthcare Life Sciences) in 0.5× TBE at 200 mA for 1 h. RNA was UV cross‐linked to the membrane and then probed for SgrS with 5′^32^P‐labeled oDH298 in ULTRAhyb‐oligo buffer (Ambion, Life Technologies) at 42^o^C. ChiX and the 5S rRNA were detected by probing the membrane with an internally ^32^P‐labeled antisense RNA [templates were generated by PCR with genomic DNA template and primers oDH234 and oDH235 (5S rRNA) or oDH308 and oDH309 (ChiX)] in ULTRAhyb buffer (Ambion, Life Technologies) at 68^o^C. Membranes were washed according to the manufacturer's instructions. Northern blots were exposed to a phosphorimager storage screen and imaged with a Storm imager (GE Healthcare).

## Supporting information

Supporting Information
