# Supplementary material for: Hfq binds directly to the ribosome‐binding site of IS 10 transposase mRNA to inhibit translation
Source: Mol Microbiol. 2015 Mar 11;96(3):633–50. doi: 10.1111/mmi.12961 (PMC5006887; doi:10.1111/mmi.12961)
Supplement: Supplementary file 1 — Supporting Information [file MMI-96-633-s001.pdf]

Supplementary Information

Hfq binds directly to the ribosome binding site of IS*10* transposase mRNA to inhibit translation

Michael J. Ellis[1], Ryan S. Trussler[1] and David B. Haniford[1#]

[1]Department of Biochemistry, University of Western Ontario, London, Ontario, Canada N6A 5C1

[#]Corresponding author: Email haniford@uwo.ca; Tel (+1) 519-661-4013

Running title: Hfq directly represses IS*10* transposase translation.

Keywords: Hfq, IS*10*/Tn*10*, ChiX sRNA, Translational regulation, Hfq titration

Table S1	List of bacterial strains and plasmids
Table S2	List of oligonucleotides
Figure S1	Hfq expression in an *hfq*$^-$ strain of *E. coli* (DBH299) used for complementation experiments
Figure S2	Mutations to the ribosome-binding site (RBS) or RNA-IN prevent Hfq binding
Figure S3	Toeprint analysis of 30S ribosome binding to *lpp* or *usg* mRNA in the presence of Hfq
Figure S4	Specificity of Hfq:RNA-IN IP and size distribution of RNA-IN molecules containing a 5'end
Figure S5	Hfq determinants for ChiX binding
Figure S6	Impact of mRNA overexpression on transposase expression

Table S1. List of bacterial strains and plasmids

Name	Description	Notes
E. coli		
DBH12	MC4100 *hfq-1*::ΩCm; Str^RCm^R	G. Storz; β-galactosidase assays and source of *hfq-1* allele for P1 transductions
DBH13	HB101 [F^- *leu*$^-$ *pro*$^-$]; Str^R	Mating out recipient[1]
DBH33	NK5830 [*recA*$^-$ *arg* $^-$Δ*lacproXIII nal*^{R} *rif*^{R}/ F' *lacpro*$^+$]	Parent strain for mating out donors[2]
DBH60	C600	Used for plasmid-λ crosses
DBH90	DBH33 λRS271 (G8, HH104); Kan^R	Chromosomal IS10-lacZ translation fusion marked with kan^R; HH104 mutation increases transposase transcription and G8 mutation increases RNA-OUT expression to maintain antisense control[3]
DBH107	MC4100; Str^R	β-galactosidase assays
DBH287	DBH107 λRS271 (G8; HH104); Str^RKan^R	Parent strain
DBH298	DBH107 λRS271 (HH104); Str^RKan^R	Chromosomal IS10-lacZ translational fusion marked with Kan^R, G8 removed by recombineering, no RNA-OUT; β-galactosidase assays (*wt*)
DBH299	DBH298 *hfq-1*::ΩCm; $Kan^RStr^RCm^R$	β-galactosidase assays (*hfq*$^-$)
DBH313	MRE600 [F^- Δ*rna*]	Steitz Lab; Strain for 30S ribosome purification
DBH331	DBH33 λDBH504 (HH104); KanR	Chromosomal IS10-Kan HH104; mating out donor strain, no RNA-OUT (*wt*)
DBH337	DBH331 *hfq-1*::ΩCm; Kan^RCm^R	Mating out donor strain and source of native RNA-IN for RIP experiments, no RNA-OUT (*hfq*$^-$)
DH5α		Cloning and plasmid propagation
**Plasmids**		
pDH858	pUC119 derived; $IS10_{1-242}$-lacZ translational fusion; Ap^R	WT IS10-lacZ translational fusion, expresses RNA-OUT
pDH866	pDH858 + R5	WT RNA-IN, RNA-OUT destabilized by R5 mutation
pDH868	pDH866 + M2	Mutated Hfq binding site 2 on RNA-IN
pDH875	pDH866 + M5	Mutated Hfq binding site 1 on

		RNA-IN
pDH700	pWKS30-p3-Hfq^{WT}; Ap^{R}	Parent plasmid; expression vector for Hfq^{WT} [4]
pDH701	pDH700-Hfq^{K56A}	Parent plasmid; expression vector for Hfq^{K56A} [4]
pDH713	pDH700-Hfq^{Y25A}	Parent plasmid; expression vector for Hfq^{Y25A} [4]
pDH874	pDH700-Hfq^{R17A}	Parent plasmid; expression vector for Hfq^{R17A}
pDH900	pACYC184 with ScaI/XmnI fragment removed; Tet^{R}Cm^{S}	Vector control for Hfq complementation experiments
pDH904	pDH900-p3-Hfq^{WT}	Expression vector for Hfq^{WT}
pDH905	pDH900-p3-Hfq^{Y25A}	Expression vector for Hfq^{Y25A}
pDH906	pDH900-p3-Hfq^{K56A}	Expression vector for Hfq^{K56A}
pDH907	pDH900-p3-Hfq^{R17A}	Expression vector for Hfq^{R17A}
pDH909	pDH900-p3-Hfq^{WT}-3xFLAG	Expression vector for Hfq^{WT}-3xFLAG
pDH763	pBR-pLlacO; Ap^{R}	Vector control for sRNA expression; S. Gottesman[5]
pDH764	pBR-pLlacO-sgrS; Ap^{R}Tet^{R}	SgrS expression; S. Gottesman[5]
pDH765	pBR-pLlacO-chiX; Ap^{R}Tet^{R}	ChiX expression; S. Gottesman[5]
pDH766	pBR-pLlacO-RybB; Ap^{R}Tet^{R}	RybB expression; S. Gottesman[5]
pDH767	pBR-pLlacO-FnrS; Ap^{R}Tet^{R}	FnrS expression; S. Gottesman[5]
pDH768	pBR-pLlacO-MicC; Ap^{R}Tet^{R}	MicC expression; S. Gottesman[5]
pDH769	pBR-pLlacO-RydC; Ap^{R}Tet^{R}	RydC expression; S. Gottesman[5]
pDH770	pBR-pLlacO-MgrR; Ap^{R}Tet^{R}	MgrR expression; S. Gottesman[5]
pDH771	pBR-pLlacO-RprA; Ap^{R}Tet^{R}	RprA expression; S. Gottesman[5]
pDH772	pBR-pLlacO-RyeB; Ap^{R}Tet^{R}	RyeB expression; S. Gottesman[5]
pDH773	pBR-pLlacO-CyaR; Ap^{R}Tet^{R}	CyaR expression; S. Gottesman[5]
pDH774	pBR-pLlacO-MicF; Ap^{R}Tet^{R}	MicF expression; S. Gottesman[5]
pDH775	pBR-pLlacO-GlmY; Ap^{R}Tet^{R}	GlmY expression; S. Gottesman[5]
pDH776	pBR-pLlacO-MicA; Ap^{R}Tet^{R}	MicA expression; S. Gottesman[5]
pDH777	pBR-pLlacO-GcvB; Ap^{R}Tet^{R}	GcvB expression; S. Gottesman[5]
pDH920	pBR-pLlacO-sodB$_{-56\ \text{to}\ +244}$; Ap^{R}Tet^{R}	*sodB* expression
pDH921	pBR-pLlacO-ptsG$_{-102\ \text{to}\ +230}$; Ap^{R}Tet^{R}	*ptsG* expression
pDH922	pBR-pLlacO-maeA$_{-73\ \text{to}\ +241}$; Ap^{R}Tet^{R}	*maeA* expression
**λ phage**		
λRS271	Source of IS10-Kan HH104 G8	R. Simons[3]
λNK1039	Encodes *his* operon	N. Kleckner; plasmid-λ crosses[6]
λDBH504	λNK1039 with IS10-Kan HH104	Product of recombination between λNK1039 and pNK1223[7]

References

1.Bolivar, F. & Backman, K. (1979). Plasmids of Escherichia coli as cloning vectors. *Methods Enzymol* **68**, 247-267.

2.Foster, T. J., Davis, M. A., Roberts, D. E., Takeshita, K. & Kleckner, N. (1981). Genetic organization of transposon Tn10. *Cell* **23**, 201-213.

3.Case, C. C., Roels, S. M., Jensen, P. D., Lee, J., Kleckner, N. & Simons, R. W. (1989). The unusual stability of the IS10 anti-sense RNA is critical for its function and is determined by the structure of its stem-domain. *EMBO J* **8**, 4297-305.

4.Ross, J. A., Wardle, S. J. & Haniford, D. B. (2010). Tn10/IS10 transposition is downregulated at the level of transposase expression by the RNA-binding protein Hfq. *Mol Microbiol* **78**, 607-21.

5.Mandin, P. & Gottesman, S. (2010). Integrating anaerobic/aerobic sensing and the general stress response through the ArcZ small RNA. *EMBO J* **29**, 3094-107.

6.Haniford, D. B., Chelouche, A. R. & Kleckner, N. (1989). A specific class of IS10 transposase mutants are blocked for target site interactions and promote formation of an excised transposon fragment. *Cell* **59**, 385-94.

7.Bender, J., Kuo, J. & Kleckner, N. (1991). Genetic evidence against intramolecular rejoining of the donor DNA molecule following IS10 transposition. *Genetics* **128**, 687-94.

Table S2. List of oligonucleotides used in this study

Name	Sequence (5' → 3')	Use
oDH502	GGATCGGAATTCCGATCTTCCCTGATGAATCCCCTAATGATTTGG	Fwd primer for making pDH858
oDH503	CCCCCCAAGCTTGGCGCCAAGTTCGGTAAGAGTGAGAGTTTTACAGTC	Rev primer for making pDH858
oDH505	GGATCGGAATTCCGATCTTCC	IS10R Fwd primer used for introducing mutations into pDH866
oDH13	CCAGTCACGACGTTGTAAAAC	LacZ reverse primer used for introducing mutations into pDH866
oDH506	CCAGTCACGACGTTGTAAAAC	Mutagenic primers for introducing R5 mutation into pDH858
oDH507	GACAAGATATGTATCCACC	
oDH498	CTCGACACCCCACACGACTCTC	Mutagenic primers for introducing M2 mutation into pDH866
oDH499	GAGAGTCGTGTGGGGTGTCGAG	
oDH508	GTCAGTCTGCGGATCACAAGATG	Mutagenic primers for introducing M5 mutation into pDH866
oDH509	CATCTTGTGATCCGCAGACTGAC	
oDH515	TAATACGACTCACTATAGGCGAAAAATCAATAACAG	Fwd primer for making IN-160 an IN^{M2}-160 ITR templates; contains T7 core promoter
oDH199	CAAGTTCGGTAAGAGTGAGAG	Rev primer for making IN-160 ITR templates; Rev primer for PCR amplification of RNA-IN cDNA
oDH510	TAATACGACTCACTATAGGCGAAAAGTCAGTCTG	Fwd primer for making IN^{M5}-160 ITR template; contains T7 core promoter
oDH511	TGTTGAGTCTCGTTTTAAGTGTA	Primer for making RNA-IN-100 cDNA (primer extension and toeprinting)
oDH513	UUUUUUU	RNA oligo; proximal site competitor
oDH514	AAAAAAAAAAAAAAAAAA	RNA oligo; distal site specific competitor
oDH480	TGTAATACGACTCACTATAGGGCTACATGGAGATTAAC	Fwd primer for making lpp ITR template; contains T7 promoter sequence
oDH482	CACGTCGTTGCTCAGCT	Reverse primer for making lpp ITR template; also used for lpp primer extension and toeprinting
oDH184	NNTCTAGANNCAGGTTGTTGGTGCTATC	Fwd primer for Hfq gene
oDH479	TTACTTGTCGTCATCGTCTTTGTAGTCCTTGTCGTCATCGTCTTTGTAGTCCTTGTCGTCATCGTCTTTGTAGTCTTCGGTTTCTTCGC	Rev primer for adding C-terminal 3xFLAG tag
oDH486	5'[Phos]GCUGAUGGCGAUGAAUGAACAC	RNA adapter; ligated to total RNA

	UGCGUUUGCUGGCUUUGAUGAAA	for reverse transcription
oDH483	GCGAAAAATCAATAATCAGAC	Fwd primer for RT-PCR of RNA-IN
oDH517	GCGAAAAGTCAGTCTGAGAC	Fwd primer for RT-PCR of RNA-IN^{M5}
oDH204	CGTGTTGTGAAATGTTGGGTTAAGT	Primer pair for RT-PCR of 16S rRNA (nt 1071-1425)
oDH205	AACCCACTCCCATGGTGTGACGGGC	
oDH518	GCCAGCTGGCGAAAGG	Primer pair for amplifying MCS of pWKS30 derived plasmids; used for introducing R17A mutation into pDH700
oDH519	TAATGCAGCTGGCACGACAGG	
oDH520	GCACTGCGTGCGGAACGTGTTCC	Mutagenic primers for introducing R17A mutation into pDH700
oDH521	GGAACACGTTCCGCACGCAGTGC	
oDH352	TTTCATCAAAGCCAGCAAACGC	RNA adapter specific primer used for cDNA synthesis
oDH353	CAAACGCAGTGTTCATTCATCGCC	RNA adapter specific nested primer used for PCR of total RNA-IN cDNA
oDH234	TAATACGACTCACTATAGGCCTGGCAGT TCCCTACTCTCG	Fwd primer for making antisense 5S rRNA (rrfA) ITR template for Northern blot; contains T7 promoter sequence
oDH235	CGGCAGTAGCGCGGTG	Reverse primer for above
oDH308	TAATACGACTCACTATAGGGAAAAAAA TGGCCAATATCGCTATTG	Fwd primer for making antisense ChiX ITR template for Northern blot; contains T7 promoter sequence
oDH309	ACACCGTCGCTTAAAGTGACG	Reverse primer for above
oDH298	CGGGTGATTTTACACCAATAC	Oligonucleotide for SgrS Northern blot
oDH528	TAATACGACTATAGACACCGTCGCTTAA AGTGAC	Fwd primer for making ChiX ITR template; contains T7 promoter sequence
oDH529	AAAAAAATGGCCAATATCGCTATTGGC CCG	Reverse primer for above
oDH554	TAATACGACTCACTATAGGCTCTGCTGT AACATTGGCAG	Fwd primer for making usg ITR template; contains T7 promoter sequence
oDH555	CCCACAGCGCCAGTTGC	Reverse primer for above; also used for toeprinting
oDH558	AATACTGACGTCATACGCACAATAAGG CTATTG	Primer pair for amplifying sodB for overexpression in pDH920
oDH559	AATACTGAATTCCAGGCAGTTCCAGTAG AAAG	
oDH560	AATACTGACGTCATAAATAAAGGGCGC	Primer pair for amplifying ptsG for

	TTAG	overexpression in pDH921
oDH561	AATACTGAATTCACGCCATCGTTATTGG	
oDH562	AATATGACGTCGCCGACGCCCTGGCG	Primer pair for amplifying maeA for overexpression in pDH922
oDH563	AATACTGAATTCGGATGTTACGCAGGTAGATG	

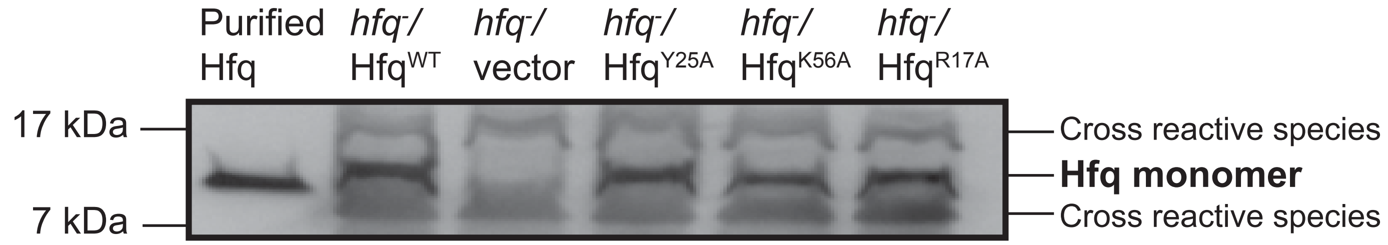

Figure S1. Hfq expression in an *hfq*$^-$ strain of *E. coli* (DBH299) used for complementation experiments. Prior to measuring β-galactosidase activity of IS*10-lacZ* (Fig. 2a), an equivalent number of cells (0.4 OD_{600}) were pelleted and frozen at -80°C. Pellets were resuspended in SDS load-mix (2% SDS, 10% glycerol, 50 mM Tris-HCl pH 6.8, 0.25% bromophenol blue and 0.8 M β-mercaptoethanol) and boiled for 5 minutes. Samples were seperated on a 14% SDS-PAGE gel and then transfered to a PVDF membrane (Roche). Hfq was detected by Western Blot using a polyclonal rabbit anti-Hfq antibody and a Pierce ECL 2 Western Blotting kit (Thermo Scientific). Purified Hfq (0.6 pmol) was loaded as a positive control.

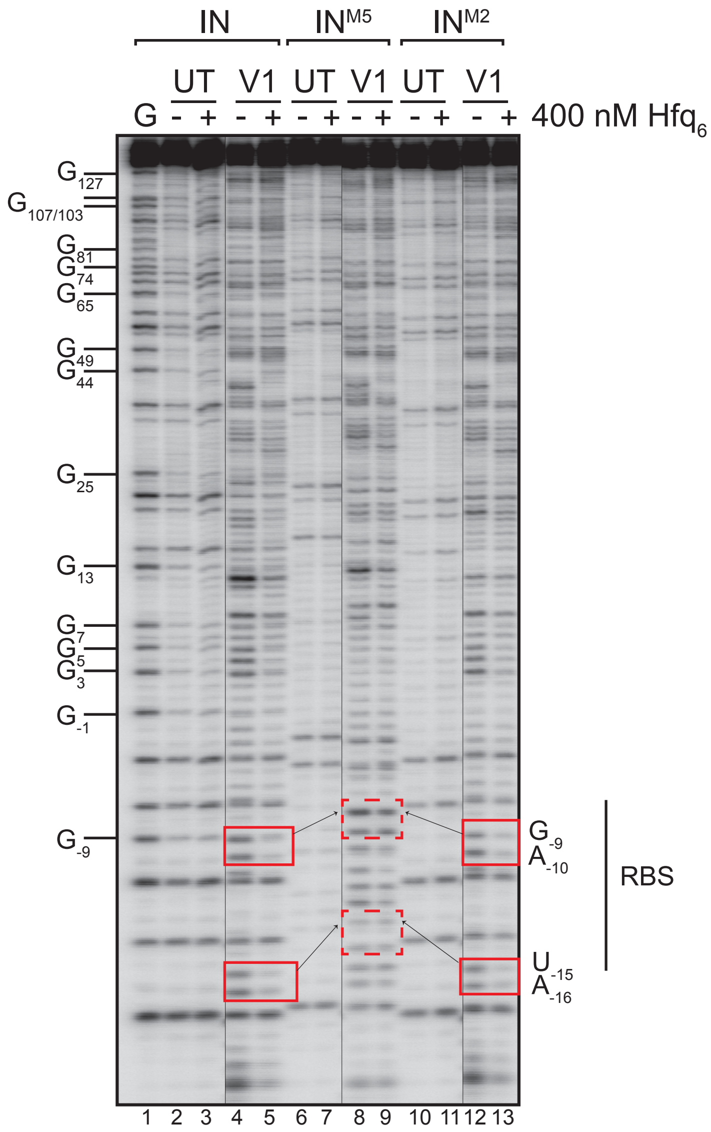

Figure S2. Mutations to the ribosome-binding site (RBS) of RNA-IN prevent Hfq binding. 5'^{32}P-labeled RNA-IN-160 (150 nM) was incubated -/+ 400 nM Hfq (hexamers) before treatment with RNase V1. Untreated controls and a RNase T1 sequencing ladder (lane 1) are shown. Nucleotide numbering is relative to the translational start. Four positions that are strongly protected from cleavage on wild-type RNA-IN (lanes 4-5) and IN^{M2} (lanes 12-13) are indicated with red boxes. The corresponding positions that are not protected on IN^{M5} are indicated with dashed red boxes. Note that the *in vitro* transcript for IN^{M5} contains an extra 5'nt, resulting in a 1nt difference in the migration of RNA fragments (indicated by black arrows). Note that certain lanes from this gel were removed for clarity (indicated by black vertical lines).

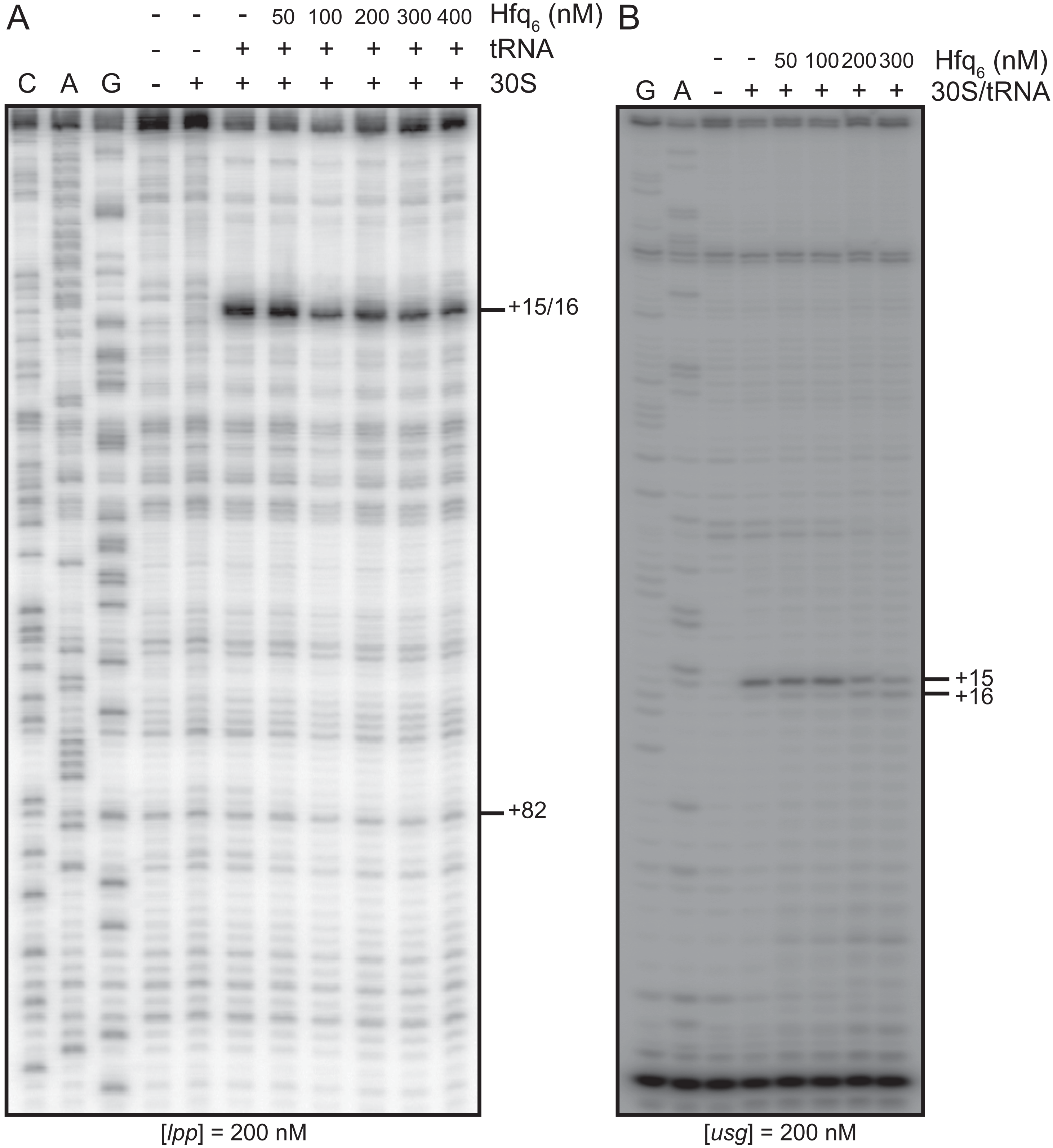

Figure S3. Toeprint analyis of 30S ribosome binding to *lpp* or *usg* mRNA in the presence of Hfq. (A) Unlabeled *lpp* (-38 to +144 relative to the translational start) or (B) *usg* (-50 to +50 relative to the translational start) mRNA was incubated with the indicated concentrations of purified Hfq before addition of the 30S ribosomal subunit (*lpp*, 270 nM; *usg*, 360 nM) and f_{Met}-tRNA (5 μM). CAG refers to sequencing lanes produced with the same RNA used for toeprinting. The toeprint signal at positions +15/+16 relative to the start codon is indicated as well as a band used to normalize loading for quantitation of the *lpp* toeprint (+82).

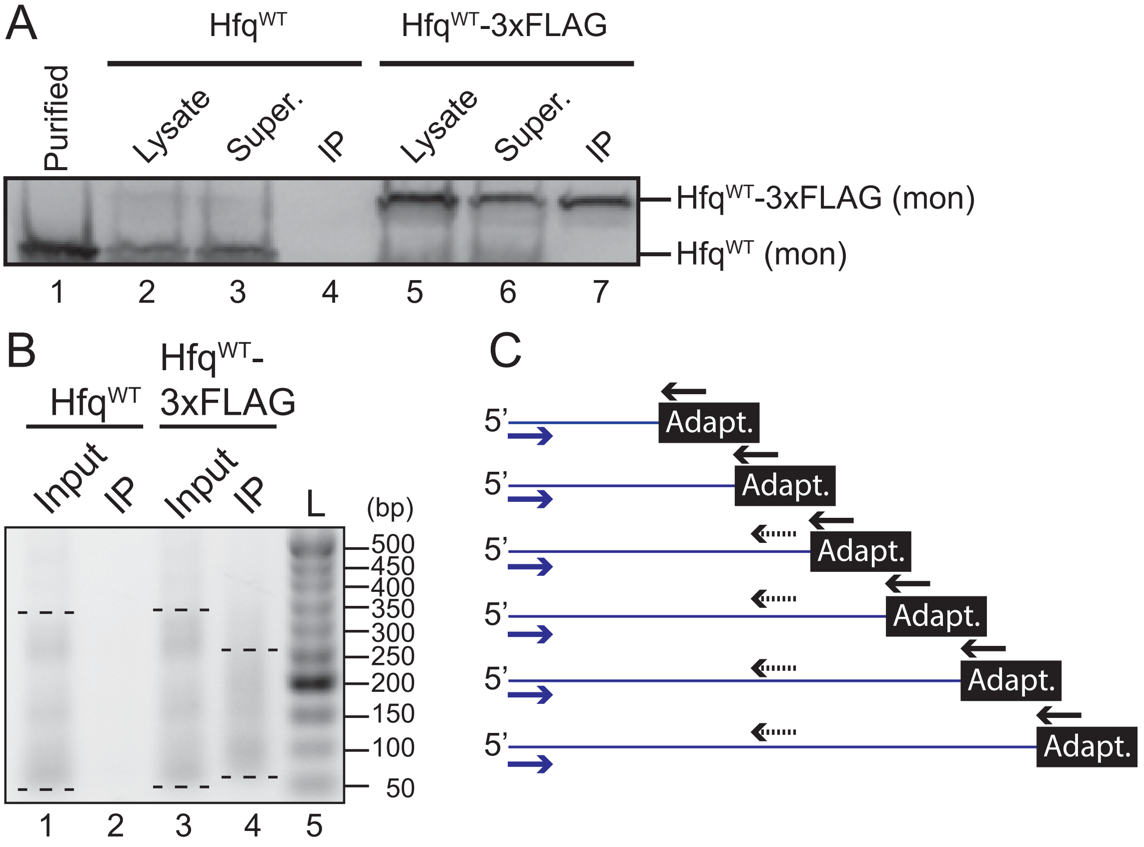

Figure S4. Specificity of Hfq:RNA-IN IP and size distribution of RNA-IN molecules containing a 5'end.
(A) Hfq western blot of fractions from Hfq^{WT} and Hfq^{WT}-3xFLAG immunoprecipitations. Aliquots of total cellular lysate (lanes 2 and 3), supernatant from IP reactions (lanes 3 and 6), or the ANTI-FLAG® M2 Magnetic Beads following washes (lanes 4 and 7) were subject to western blot as described in Figure S1. Purified Hfq (0.15 pmol) was loaded as a positive control (lane 1). The tagged and untagged Hfq monomers are indicated.
(B) cDNA from total RNA (lanes 1 and 3) or IP reactions (lanes 2 and 5) was amplified by 24 cycles of PCR using an RNA-IN specific forward primer and a nested RNA Adapter specific reverse primer. PCR products were then resolved on a 2% Agarose/TBE gel and stained with EtBr. A DNA ladder (lane 5) was used to determine the size distribution of PCR proucts which is indicated with dashed horizontal lines.
(C) Schematic of RT-PCR used for Hfq IP. Total RNA from the RIP was ligated to an RNA adapter (black box). An adapter specific primer was for cDNA synthesis, yielding a mixed population of RNA species. The size distribution of RNA-IN molecules with an intact 5'end (B) was determined by amplifying PCR with an RNA-IN specific forward primer (blue arrow) that anneals to the extreme 5'end and a nested adapter specific primer (black arrow). PCR shown in Fig. 7 used an RNA-IN specific forward primer (blue) and an RNA-IN specific reverse primer (dashed black arrow) to amplify the first 160-nt of RNA-IN. Accordingly, the RNA-IN signal shown in Fig. 7 originates from RNA-IN molecules containing at least the first 160-nt of RNA-IN.

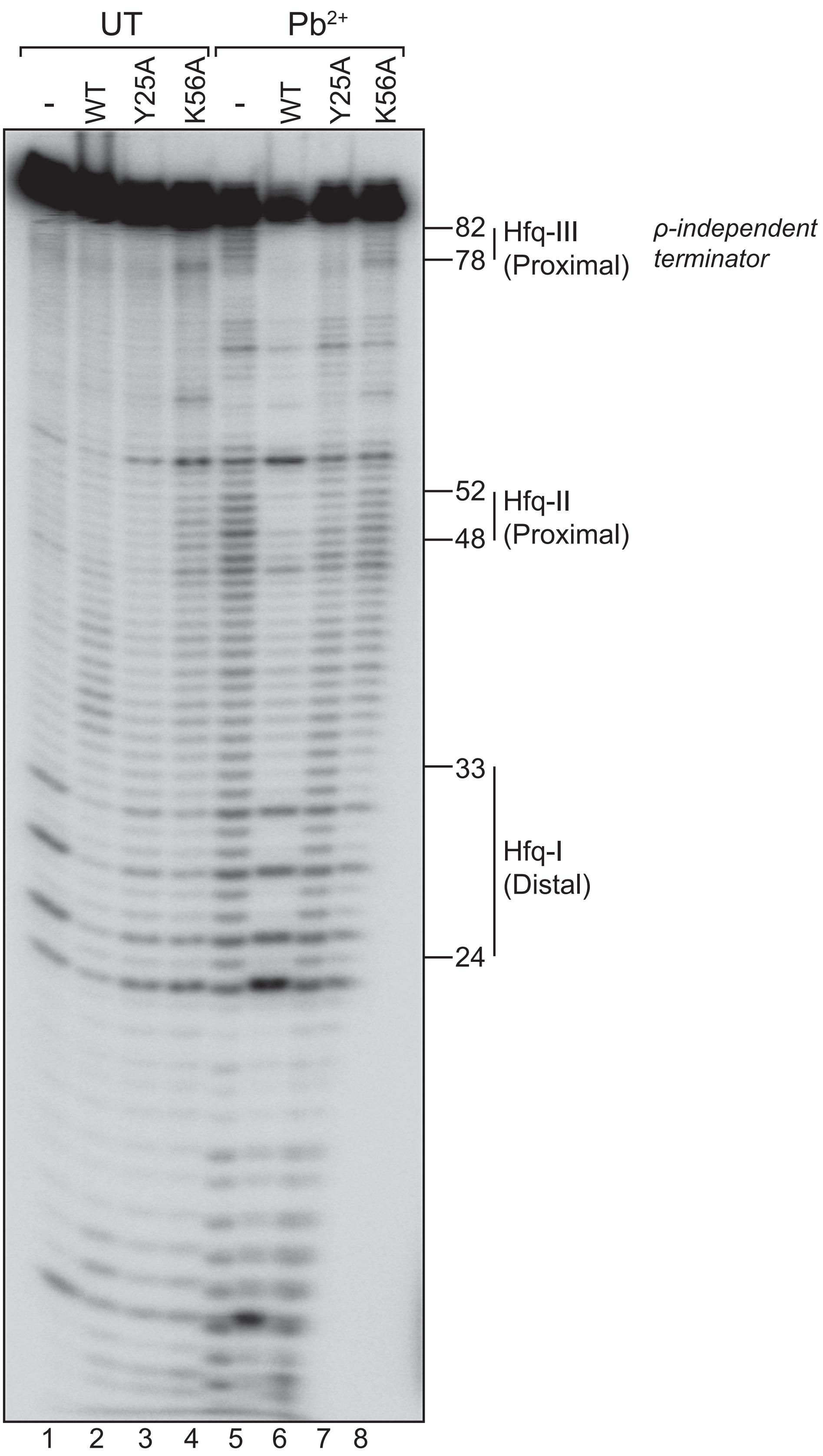

Figure S5. Hfq determinants for ChiX binding. 5'^{32}P-labeled ChiX (140 nM) was incubated with WT and mutant variants of his-tagged Hfq (380 nM) before limited cleavage with Pb^{2+}(lanes 5-8). Untreated controls are shown (lanes 1-4). The location of the two Hfq-binding sites identified in Fig. 9C (Hfq-I and Hfq-II) are highlighted along with a third site (Hfq-III) that corresponds to the polyU tail after the ρ-independent transcriptional terminator. Sites are defined as interacting with the distal surface if they are protected by $Hfq^{WT}$and Hfq^{K56A} but not Hfq^{Y25A}. Likewise, sites that show reduced protection with Hfq^{K56A} compared to Hfq^{WT}/Hfq^{Y25A} are defined as interacting with the proximal surface of Hfq.

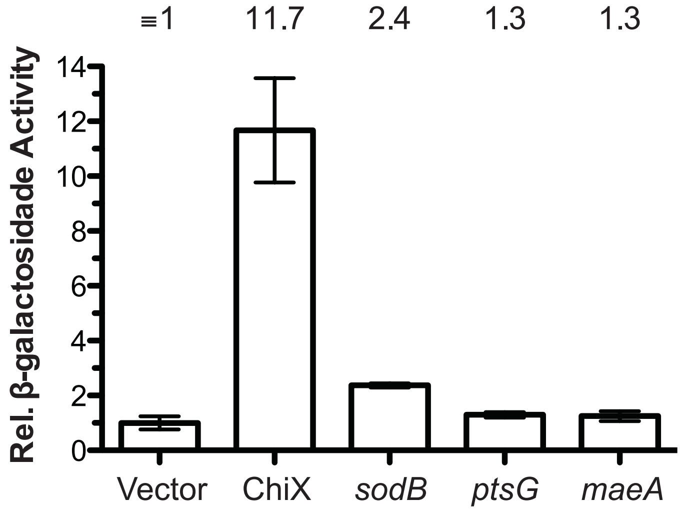

Figure S6. Impact of mRNA overexpression on transposase expression. *hfq*$^+$ cells containing the chromosomal IS*10*$_{1\text{-}337}$-*lacZ* translational fusion (DBH298) were transformed with a plasmid expressing ChiX (pDH765), *sodB*$_{-56 \text{ to } +244}$ (pDH920), *ptsG*$_{-102 \text{ to } +230}$ (pDH921), *maeA*$_{-73 \text{ to } 241}$ (pDH922) or a vector control (pDH763). Transformants were grown to mid-exponential phase in LB media and β-galactosidase activity was measured by the Miller assay. Error bars show the standard error on the mean for two independent experiments (n=6) and the relative expression is shown above the graph, where transposase-*lacZ* expression in the presence of vector was set to 1.
